# The Oxidative Extraction of Starch from Chestnut (*Castanea sativa* Mill.) Byproducts: A Valorization Strategy for a Sustainable Food Industry

**DOI:** 10.3390/polym18030356

**Published:** 2026-01-28

**Authors:** Luís Moreira, Juliana Milheiro, Fernanda Cosme, Fernando M. Nunes

**Affiliations:** 1 Food and Wine Chemistry Lab., CQ-VR—Chemistry Research Centre—Vila Real, 5000-801 Vila Real, Portugal; luismoreira@utad.pt (L.M.); fcosme@utad.pt (F.C.); 2 Department of Biology and Environment, School of Life Sciences and Environment, University of Trás-os-Montes and Alto Douro, 5000-801 Vila Real, Portugal; 3 Chemistry Department, School of Life Sciences and Environment, University of Trás-os-Montes and Alto Douro, 5000-801 Vila Real, Portugal

**Keywords:** chestnut byproducts, starch recovery, hypochlorite, oxidation, sustainability

## Abstract

Global chestnut production is rising. However, the Portuguese chestnut industry still experiences annual post-harvest losses, largely due to microbial spoilage. Recovering high-value starch from spoiled chestnuts offers a promising strategy to reduce waste and increase economic returns. Yet, starch extracted from spoiled kernels is typically dark brown, limiting its industrial applications. This study aimed to enhance the sustainability of the chestnut sector by converting industrial byproducts into useful ingredients. We evaluated whether hypochlorite-mediated oxidative extraction at pHs around 8 and 12 could produce starch with functional properties suitable for industrial applications. Both native and bleached starches showed similar lightness (L* 84–91), though a slight yellow hue remained (ΔE* 12–19). The degree of crystallinity was higher in bleached starches (13–16%) while preserving the characteristic C_B_-type crystalline pattern of native chestnut starch. The degree of oxidation was 0.88% and 0.43% for bleached starches isolated at pHs 8 and 12, respectively. Starch bleached at pH 8 exhibited moderate viscosity (breakdown 0.103) and greater swelling capacity at 50 °C than corn starch. In contrast, extraction under alkaline conditions markedly reduced gelatinization and retrogradation performance. Therefore, oxidative extraction at middle pH proved to be the most effective method for recovering functional starch from spoiled chestnuts.

## 1. Introduction

Sustainability in the food industry and agricultural sectors is essential. These systems are major consumers of land, water, and energy and thus have a substantial environmental footprint. A transition from linear production models toward circular economy frameworks supports more efficient resource use, waste reduction, and improved environmental outcomes. The circular economy emphasizes keeping biological materials in use for as long as possible. It also recovers valuable compounds from agricultural residues, helping to reduce dependency on virgin raw materials and decrease pollution [1,2].

A crucial component of circularity is the valorization of byproducts. Materials that were previously discarded, such as fruit peels, seeds, pomace, and processing residues, can be made into valuable secondary raw materials. These byproducts are rich in bioactive compounds including polyphenols, carotenoids, dietary fibers, vitamins, essential oils, and enzymes. They can be recovered and used in food, pharmaceutical, cosmetic, or packaging applications [3]. By valorizing agricultural and food processing residues, it is possible not only to reduce waste disposal costs and mitigate environmental pollution but also to generate economic value and create new product streams [1,4].

Global chestnut production has been steadily increasing, reaching approximately 3.63 million tons in 2024 [5]. Nevertheless, in Portugal, the Portuguese Chestnut Association [6] estimates annual losses of 15% to 30%, depending on climatic conditions and chestnut management practices. Post-harvest contamination during harvesting, transportation, and storage further contributes to this decline. Beyond the economic impact, the high proportion of spoiled chestnuts poses additional challenges. Much of the affected produce is discarded or sold at very low market value for animal feed, yielding minimal profit. This situation underscores the need for effective strategies to enhance both the cost-efficiency and sustainability of chestnut production.

In this context, extracting high-value starch from spoiled chestnuts offers a promising response to address several critical challenges in the agro-industrial sector. Rather than being discarded, spoiled chestnuts can be reintroduced into the value chain as a resource with significant economic and environmental benefits. This approach not only supports the production of a valuable ingredient but also contributes to more sustainable resource management, reducing waste and promoting circularity within modern industrial practices [2].

Starch is a highly versatile material used across a wide range of industries, including animal feed production [7], the paper industry [8], and, most prominently, the food industry. In food applications, starch functions as a thickener, stabilizer, gelling agent, syneresis controller, fat replacer, and emulsifier [9]. Resistant starch can also contribute as dietary fiber [10,11]. Furthermore, the development of starch-based films for packaging has gained significant attention in recent years. These films offer a practical and biodegradable alternative that supports environmental sustainability [12,13].

Native chestnut starch can be isolated with high purity using enzymatic extraction methods [14,15]. However, more cost-effective methods have been shown to produce starches of comparable or even superior quality. These approaches typically involve steeping disintegrated or milled chestnut kernels in sodium bisulfite [16,17] or sodium hydroxide solutions [14,17,18]. Such techniques have successfully isolated chestnut starch with B-type [16,17] or C-type crystalline conformations [14,19,20], respectively. Nonetheless, to the best of our knowledge, comprehensive studies evaluating how these steeping solutions influence the crystallinity and functional properties (such as thermal stability and pasting behavior) of starch isolated from *Castanea sativa* Mill. are still lacking.

Furthermore, a significant challenge in isolating starch from deteriorated sources is the dark coloration of the final product. This limits its suitability for applications requiring high transparency or lightness [21,22]. As a result, bleaching becomes a crucial step in producing a value-added starch product. Among the available treatments, hypochlorite oxidation is one of the most commonly used methods for starch bleaching [23,24]. However, this treatment can also alter the chemical structure of starch by oxidizing hydroxyl groups. This may affect key functional properties such as thermal stability, viscosity, solubility, and film-forming capacity [23,25,26,27,28]. Therefore, a thorough characterization of this product is essential to assess its potential applications in the food industry.

This pioneering study aims to enhance the sustainability of the chestnut sector by adding value to industrial byproducts. This work represents, to the best of our knowledge, the first systematic evaluation of hypochlorite-mediated oxidative extraction applied to starch recovery from spoiled chestnut kernels. Specifically, we investigate whether oxidative extraction at middle and alkaline pH levels can yield starch from spoiled chestnut kernels with functional properties (e.g., viscosity and thickening capacity, thermal stability, water-binding and moisture retention ability), suitable for applications in the food industry.

To this end, hypochlorite-treated starches were directly compared with native starches isolated from conforming chestnuts under the same pH conditions. This design allows for a direct comparison between starches derived from conforming and spoiled raw materials. Based on the findings, recommendations are made regarding the most suitable extraction method for potential large-scale production.

## 2. Materials and Methods

### 2.1. Materials

Chestnut (*Castanea sativa* Mill.) cultivars Longal, Judia, and Martaínha were harvested during the chestnut season (November 2021) in the Trás-os-Montes region of Portugal and supplied in frozen form by AgroAguiar—Agroindústria S.A. (Vila Pouca de Aguiar, Portugal). The chestnut byproducts used in this study consisted of spoiled kernels, with or without residual shell fragments remaining after mechanical shelling. Commercial-grade starches from corn (Sigma, St. Louis, MO, USA, S4126) and potato (Cimarrom, Frutogal, Barcelos, Portugal) were used as reference standards. A commercial bleach solution containing 5% active chlorine was purchased from local retailers. All other chemicals used were of analytical grade.

### 2.2. Starch Isolation

Starches were isolated from both conforming and spoiled chestnuts using either the sodium bisulphite method described by Cruz et al. [16] or the alkaline extraction method described by Zhang et al. [29] with necessary adaptations. Thawed chestnuts (500 g), both conforming and spoiled, were chopped and homogenized in 2.5 L of either 25 mM NaHSO_3_ or 0.2% (*w*/*v*) NaOH solution. Conforming chestnut flour was also used to extract starch using either NaHSO_3_ or NaOH. The mixtures were incubated at room temperature for 24 h with occasional shaking. Bleached starches were prepared by adding commercial bleach (32% *v*/*v* corresponding to 1.65% active chlorine) to the slurries of spoiled chestnuts steeped in the respective NaHSO_3_ or NaOH solutions. The slurries were then decanted and washed multiple times with tap water until the supernatant became clear. Subsequently, the slurries were transferred to a vibratory sieve shaker (AS 200 Control, Retsch, Verder Scientific, Haan, Germany) equipped with 200, 100, and 53 µm meshes to separate insoluble debris from starch. Table S1 describes the flour and starch samples used in each experiment. Finally, the starch slurries were then frozen, freeze-dried, milled, and sieved through a 53 µm mesh. Table 1 describes the eight different starch samples prepared for this study. The starches were finally weighted to determine the extraction yield (%) according to Equation (1):

(1) $$\mathrm{Yield}\;(\%)=\frac{m_{\mathrm{starch}}\left(g\right)}{m_{\mathrm{chestnuts}}\left(g\right)}\times 100$$

where m_starch_ and m_chestnuts_ are the masses (g) of the freeze-dried starch and freeze-dried chestnuts, respectively.

The moisture content of chestnuts was determined by measuring the weight loss after freeze-drying. To assess the moisture content of chestnut flours and starches, samples were first equilibrated at 50% relative humidity, after which their water content was measured using thermogravimetric analysis (TGA).

**Table 1:** CIELab color coordinates of chestnut starches extracted with NaHSO_3_ or NaOH, with and without bleaching treatment. Flours from conforming and spoiled chestnuts were included for comparison, and commercial corn starch is used as reference.

Samples	L*	a*	b*	C*	ΔE*
Commercial corn starch	99.23 ± 0.01 ^a^	−0.74 ± 0.03 ^h^	3.55 ± 0.02 ^h^	3.63 ± 0.02 ^h^	n.a.
(2) Conforming chestnut flour	89.55 ± 0.23 ^c^	−2.01 ± 0.09 ^i^	17.36 ± 0.28 ^a^	17.47 ± 0.28 ^a^	16.91 ± 0.20 ^e^
(1) Spoiled chestnut flour	71.98 ± 0.04 ^h^	1.66 ± 0.02 ^c^	11.93 ± 0.10 ^e^	12.04 ± 0.10 ^e^	28.61 ± 0.01 ^b^
(3) Conforming chestnut starch_NaHSO_3_	89.45 ± 0.57 ^d^	−0.39 ± 0.00 ^f,g^	10.90 ± 0.09 ^f^	10.91 ± 0.09 ^f^	12.24 ± 0.41 ^h^
Conforming chestnut starch_NaHSO_3_ (from flour)	90.12 ± 0.12 ^c^	0.42 ± 0.03 ^e^	6.64 ± 0.05 ^g^	6.66 ± 0.05 ^g^	9.69 ± 0.12 ^j^
(5) Spoiled_chestnut_starch_NaHSO_3_	74.42 ± 0.07 ^g^	2.11 ± 0.00 ^b^	14.07 ± 0.01 ^d^	14.23 ± 0.01 ^c^	27.10 ± 0.07 ^c^
(7) Spoiled chestnut starch NaHSO_3_ + Bleach	83.82 ± 0.03 ^f^	0.91 ± 0.01 ^d^	15.30 ± 0.01 ^c^	15.33 ± 0.01 ^b^	19.45 ± 0.03 ^d^
(4) Conforming chestnut starch_NaOH	87.61 ± 0.17 ^e^	−0.66 ± 0.01 ^h^	13.79 ± 0.14 ^d^	13.81 ± 0.14 ^d^	15.49 ± 0.16 ^f^
Conforming chestnut starch_NaOH (from flour)	89.82 ± 0.05 ^c^	−0.31 ± 0.01 ^f^	11.12 ± 0.18 ^d^	11.13 ± 0.18 ^d^	12.09 ± 0.15 ^h,i^
(6) Spoiled_chestnut_starch_NaOH	69.90 ± 0.13 ^i^	4.44 ± 0.02 ^a^	16.59 ± 0.04 ^b^	17.17 ± 0.04 ^a^	32.51 ± 0.13 ^a^
(8) Spoiled chestnut starch NaOH + Bleach	90.89 ± 0.03 ^b^	−0.43 ± 0.02 ^g^	15.02 ± 0.11 ^c^	15.03 ± 0.11 ^b^	14.18 ± 0.11 ^g^

**Figure 1:**
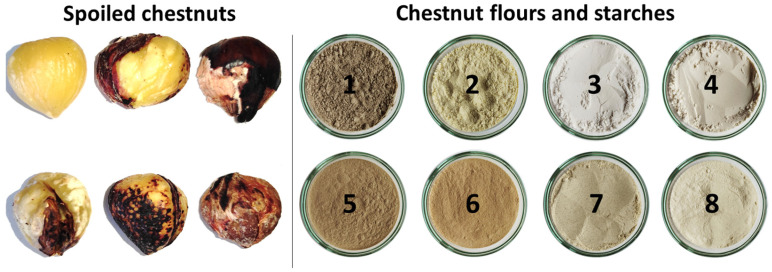
Representative pictures of non-conforming and spoiled chestnut kernels (**right**). Representative images of the following: spoiled chestnut flour (1), conforming chestnut flour (2), conforming chestnut starch_NaHSO_3_ (3), conforming chestnut starch_NaOH (4), spoiled _chestnut_starch_NaHSO_3_ (5), spoiled _chestnut_starch_NaOH (6), spoiled chestnut starch NaHSO_3_ + Bleach (7), and spoiled chestnut starch NaOH + Bleach (8) (**left**).

L*—lightness; a*—redness–greenness; b*—yellowness–blueness; C*—chroma; ΔE*—total color difference; n.a.—not applicable. Values are expressed as the means of triplicates ± standard deviations. Data were subject to a one-way analysis of variance (ANOVA) followed by Tukey’s post hoc multiple-comparison test. Means within the same column followed by different superscript letters differ significantly (*p* < 0.05). The numbers preceding the names indicate the corresponding samples presented in Figure 1.

### 2.3. Color Evaluation

Chestnut starch color was measured using a Chroma Meter CR-400 (Konica Minolta, Osaka, Japan). CIELab coordinates were obtained under a C/2° illuminant/observer reference system. The instrument was calibrated using a white standard plate (L* = 94.53; a* = −0.24; b* = 4.24), which was also used as the background for film measurements. For each sample, L*, a*, and b* values were calculated as the average of three measurements. Commercial corn starch (Sigma, S-4126) was used as the reference starch. Chroma values were then calculated according to Equation (2):

(2) $$C^{*}=\sqrt{a^{*2}+b^{*2}}$$

ΔE*, the total color difference, was calculated according to Equation (3):

(3) $$\Delta E^{*}=\sqrt{{\left(\Delta L*\right)}^{2}+{\left(\Delta a*\right)}^{2}+{\left(\Delta b*\right)}^{2}}$$

where ΔL*, Δa*, and Δb* represent the differences in color parameter values between the chestnut starch and the reference corn starch. Color measurements were performed in triplicate on 11 samples, namely the following: commercial corn starch, conforming chestnut flour, spoiled chestnut flour, conforming chestnut starch_NaHSO_3_, conforming chestnut starch_NaHSO_3_ (from flour), spoiled _chestnut_starch_NaHSO_3_, spoiled chestnut starch NaHSO_3_ + Bleach, conforming chestnut starch_NaOH, conforming chestnut starch_NaOH (from flour), spoiled chestnut_starch_NaOH, and spoiled chestnut starch NaOH + Bleach.

### 2.4. Determination of Granule Structure and Size of Chestnut Starch by Scanning Electron Microscopy (SEM)

The morphology of chestnut starch granules was examined using an FEI Quanta 400 Scanning Electron Microscope (FEI Company, Hillsboro, OR, USA) equipped with a W filament and operated in environmental mode at 6 mbar with a Large Field Detector (LFD). Starch samples were suspended in water for 24 h with occasional stirring. A single drop of the suspension was applied onto carbon adhesive and allowed to dry at room temperature. Imaging was performed at an accelerating voltage of 30 kV. Granule size measurements were carried out using ImageJ2 software (National Institutes of Health, USA). SEM analysis was performed on 4 samples, namely the following: spoiled _chestnut_starch_NaHSO_3_, spoiled chestnut starch NaHSO_3_ + Bleach, spoiled chestnut_starch_NaOH, and spoiled chestnut starch NaOH + Bleach.

### 2.5. Thermogravimetric Analysis (TGA)

Thermogravimetric analysis (TGA) was performed using a NETZSCH STA 449F3 Jupiter thermal analyzer (Selb, Germany). Samples of starch and plastic films (5–10 mg) were placed in alumina crucibles and heated from 25 °C to 700 °C at a rate of 10 °C min^−1^ under a nitrogen atmosphere (flow rate 50 mL min^−1^) to prevent thermo-oxidative degradation. The baseline curve obtained from an empty alumina crucible was subtracted from each sample curve. Thermogravimetric (TG) and derivative thermogravimetric (DTG) data were processed using NETZSCH Proteus software (Version 6.1.0). TGA was performed in triplicate on 8 samples, namely the following: conforming chestnut flour, spoiled chestnut flour, conforming_chestnut_starch_NaHSO_3_, conforming_chestnut_starch_NaOH, spoiled chestnut starch NaHSO_3_, spoiled chestnut starch NaOH, spoiled chestnut starch NaHSO_3_ + Bleach, and spoiled chestnut starch NaOH + Bleach.

### 2.6. Aflatoxin Analysis

Total aflatoxin levels in spoiled chestnut flour and in starches obtained by NaHSO_3_ + Bleach and NaOH + Bleach treatments were determined using the RIDA^®^QUICK Aflatoxin RQS kit (R-Biopharm, Darmstadt, Germany), following the manufacturer’s instructions. Briefly, 10 g of each sample was weighed and extracted with 20 mL of 70% (*v*/*v*) methanol by shaking for 5 min at room temperature. The extracts were then centrifuged for 1 min at 2000× *g*, and 100 µL of the resulting supernatant was mixed with 200 µL of the provided mobile solvent. Subsequently, 100 µL of the diluted extract was applied to the test strip and incubated for 3 min at room temperature. After exactly 3 min, the test strips were analyzed using the RIDA^®^SMART App (Version RSA_4.06_Rbio). An aflatoxin test was performed in triplicate for each sample.

### 2.7. Total Starch and Resistant Starch Content

Total starch and resistant starch contents were determined according to AACC method 76–31.01 using the K-TSTA kit (Megazyme International Ireland Co., Ltd., Wicklow, Ireland), with modifications. To verify the potential presence of resistant starch, two sample preparation methods were applied: (a) dispersion in sodium acetate buffer (100 mM, pH 5) with 5 mM calcium chloride and (b) pre-dispersion in 80% ethanol and cold 1.7 M NaOH, followed by solubilization in the buffer. Both treatments were subjected to enzymatic hydrolysis with α-amylase and amyloglucosidase, according to the manufacturer’s protocol. Glucose quantification was carried out using high-performance anion-exchange chromatography with pulsed amperometric detection (HPAEC-PAD) instead of the kit’s colorimetric method. Analyses were performed on a Dionex ICS 3000 system (Dionex, Sunnyvale, CA, USA) equipped with Carbopac PA-100 Guard and Carbopac PA-100 (250 mm × 4 mm) columns. Following sample injection (0.3 mL), elution was performed with a linear gradient (0–65 min) from 100% eluent A (150 mM NaOH containing 2 mM Ba(OH)_2_) to 100% eluent B (150 mM NaOH and 400 mM sodium acetate containing 2 mM Ba(OH)_2_). After each run, the column was washed with 500 mM NaOH containing 2 mM Ba(OH)_2_ for 10 min and re-equilibrated for 15 min with eluent A. The flow rate was 1.0 mL·min^−1^, and the column temperature was maintained at 35 °C. The electrochemical detector consisted of a Au working electrode, Ag/AgCl reference electrode, and Ti counter electrode. Applied pulse potentials and durations were as follows: E_1_ = 0.05 V (t_1_ = 200 ms), E_2_ = 0.75 V (t_2_ = 200 ms), and E_3_ = −0.15 V (t_3_ = 400 ms). The sampling rate was set to 1 Hz, with an integration window of 0.2–0.4 s. Peak areas were corrected for molar PAD detector responses. Starch content was determined in triplicate on 4 samples, namely the following: conforming_chestnut_starch_NaHSO_3_, conforming_chestnut_starch_NaOH, spoiled chestnut starch NaHSO_3_ + Bleach, and spoiled chestnut starch NaOH + Bleach.

### 2.8. Determination of Carboxyl Group Content in Chestnut Starches

The carboxyl group content in the starches was determined following the methods of Yu et al. [30] and Dang et al. [31], with modifications. Briefly, freeze-dried starch samples (10.0 g) were mixed with 100 mL of 0.1 M HCl and stirred for 30 min. The resulting slurries were centrifuged at 3000× *g* for 5 min to remove the HCl solution, then washed once with 0.1 M HCl and three times with Milli-Q water to eliminate cations that could interfere with the analysis. After freeze-drying, a 2 g portion of each starch sample was gelatinized in 150 mL of Milli-Q water and cooled to 30 °C. Subsequently, 20 drops of phenolphthalein indicator (1 g phenolphthalein in 100 mL of 60% ethanol) were added, and the solution was titrated with 0.01 M NaOH, recording the volume of NaOH consumed (V_oxidized_). A blank sample was prepared in the same manner using native starch isolated either with NaHSO_3_ or NaOH (V_native_). The carboxyl content (%) was then calculated following Equation (4):

(4) $$\mathrm{Carboxyl}\;\mathrm{content}\;(\%)\;=\;\frac{C_{\mathrm{NaOH}}\times \left(V_{\mathrm{oxidized}}-V_{\mathrm{native}}\right)}{1000\times 3\times m/M}\times 100$$

where C_NaOH_ (M) is the concentration of NaOH standard solution, V_oxidized_ (mL) is the volume of NaOH solution used to titrate the oxidized starch sample, V_native_ (mL) is the volume of NaOH solution used to titrate the native starch sample, m (g) is the weight of the starch samples, M is the molar mass of an anhydroglucose unit (162 g/mol), and 3 represents the number of hydroxy groups in each anhydroglucose unit. Carboxyl content was determined in triplicate on 2 samples, namely the following: spoiled chestnut starch NaHSO_3_ + Bleach and spoiled chestnut starch NaOH + Bleach.

### 2.9. Determination of Carbonyl Group Content in Chestnut Starches

The carbonyl group content of the starch samples was determined using the method described by Yu et al. [30] and Dang et al. [31], with slight modifications. Freeze-dried starch (2.0 g) was gelatinized in 100 mL of distilled water under continuous stirring, then cooled to 40 °C and adjusted to pH 3.2. Subsequently, 16 mL of hydroxylamine reagent (prepared by dissolving 25 g of hydroxylamine hydrochloride in 100 mL of 0.5 mol/L NaOH and diluting to 500 mL) was added. The flasks were sealed and incubated in a water bath at 40 °C for 4 h with occasional stirring. The unreacted hydroxylamine in the starch solution was titrated with a 0.01 M HCl standard solution until a pH of 3.2 was reached, and the volume of HCl consumed (V_oxidized_) was recorded. A blank was prepared in the same manner using native starch isolated with NaHSO_3_ or NaOH (V_native_). The carbonyl content (%) was calculated according to Equation (5):

(5) $$\mathrm{Carbonyl}\;\mathrm{content}\;(\%)\;=\;\frac{C_{\mathrm{HCl}}\times (V_{\mathrm{native}}-V_{\mathrm{oxidized}})}{1000\times 3\times m/M}\times 100$$

where C_HCl_ (M) is the concentration of the HCl standard solution, V_native_ (mL) is the volume of HCl solution used to titrate the native starch sample, V_oxidized_ (mL) is the volume of HCl standard solution used to titrate the oxidized starch sample, m (g) is the mass of the starch samples, M is the molar mass of an anhydroglucose unit (162 g/mol), and 3 represents the number of hydroxy groups in an anhydroglucose unit. Carbonyl content was determined in triplicate on 2 samples, namely the following: spoiled chestnut starch NaHSO_3_ + Bleach and spoiled chestnut starch NaOH + Bleach.

### 2.10. Characterization of Bleached Starches Using Electrospray Ionization–Mass Spectrometry (ESI-MS)

Starch samples (100 mg) were suspended in 10 mL of 100 mM sodium acetate buffer (pH 5.0) containing 5 mM calcium chloride. A total of 100 μL of α-amylase (2500 U/mL, *Bacillus* sp., Sigma-Aldrich) and 200 μL of pullulanase (1000 U/mL, *Bacillus acidopullulyticus*, Sigma-Aldrich) were added to each suspension. The tubes were sealed and incubated at 40 °C for 24 h with occasional shaking. After incubation, the samples were cooled to room temperature and stored at −20 °C until further use. Upon thawing and homogenization, 0.2 mL of each sample was diluted in 0.8 mL of methanol containing 1% (*v*/*v*) formic acid [32]. The diluted samples were directly infused into an ESI-MS LTQ XL (Thermo Scientific, Waltham, MA, USA), and the resulting spectra were analyzed using Thermo Xcalibur software (Version 4.2.47). ESI-MS was performed in triplicate on 4 samples, namely the following: conforming_chestnut_starch_NaHSO_3_, conforming_chestnut_starch_NaOH, spoiled chestnut starch NaHSO_3_ + Bleach, and spoiled chestnut starch NaOH + Bleach.

### 2.11. High-Performance Size Exclusion Chromatography (HPSEC) of Starch

To compare the molecular weight profile, starch samples (25 mg) were gelatinized in 2 mL of distilled water at 90 °C for 30 min with constant agitation. Then, 0.1 mL of 2 M NaOH solution was added, and the mixture was vortexed for 1 min and then sonicated for 8 s at 75% amplitude. The solubilized starch was neutralized with 0.1 mL of 2 M HCl and centrifuged at 4000 rpm for 10 min. The supernatant was collected and diluted with the eluent at a 1:2 ratio. The resulting solution (100 μL injection volume) was analyzed by high-performance size exclusion chromatography (Dionex UltiMate 3000, Thermo Scientific, Waltham, MA, USA) equipped with an OHpak SB804 HQ column (300 mm × 8 mm × 10 μm, Shodex, Tokyo, Japan). The eluent consisted of 100 mM sodium nitrate containing 0.02% sodium azide, delivered at a flow rate of 0.45 mL/min at 70 °C. The eluant was continuously monitored using an LKB 2142 refractometer (Bromma, Stockholm, Sweden). The exclusion and total volume of the column were determined by using blue dextran (1 mg/mL, 100 μL injection volume) and glucose (10 mg/mL, 50 μL injection volume), respectively. HPSEC was performed in triplicate on 6 samples, namely the following: conforming_chestnut_starch_NaHSO_3_, conforming_chestnut_starch_NaOH, spoiled chestnut starch NaHSO_3_, spoiled chestnut starch NaOH, spoiled chestnut starch NaHSO_3_ + Bleach, and spoiled chestnut starch NaOH + Bleach.

### 2.12. X-Ray Diffraction (XRD) and Relative Crystallinity

X-ray diffraction (XRD) measurements were performed at room temperature using a Panalytical X’Pert Pro MPD diffractometer (Almelo, The Netherlands), equipped with an X’Celerator detector and a secondary monochromator in θ/2θ Bragg–Brentano geometry. Data were collected with CuKα radiation (λα1 = 1.54060 Å; λα2 = 1.54443 Å) over a 2θ angular range of 5–50° with a scan step size of 0.017° and a counting time of 100 s per step. Prior to analysis, starch granules and plastic films were equilibrated at 50% relative humidity. Crystalline and amorphous areas were determined using OriginPro 8.5 software following the procedure described by Huang et al. [33]. The relative degree of crystallinity (%Cr) of the starch granules was determined using Equation (6):

(6) $$\%Cr=\frac{Ac}{Ac+Aa}\;\times 100$$

where Ac represents the crystalline area, and Aa represents the amorphous area in the X-ray diffractogram. XRD was performed on 8 samples, namely the following: conforming chestnut flour, spoiled chestnut flour, conforming_chestnut_starch_NaHSO_3_, conforming_chestnut_starch_NaOH, spoiled chestnut starch NaHSO_3_, spoiled chestnut starch NaOH, spoiled chestnut starch NaHSO_3_ + Bleach, and spoiled chestnut starch NaOH + Bleach.

### 2.13. Attenuated Total Reflectance-Fourier Transform Infrared Spectroscopy (ATR-FTIR)

The short-range molecular order of starches and plastic films was analyzed by Attenuated Total Reflectance–Fourier Transform Infrared (ATR-FTIR) spectroscopy. Compacted starch samples were scanned from 4000 to 400 cm^−1^ using a Shimadzu IRAffinity-1S spectrometer (Kyoto, Japan) equipped with an attenuated total reflection diamond crystal accessory. Each spectrum was collected from 128 scans at a resolution of 2 cm^−1^, then baseline-corrected (4000–400 cm^−1^) and normalized between 0 and 1. ATR-FTIR was performed in triplicate on 4 samples, namely the following: conforming_chestnut_starch_NaHSO_3_, conforming_chestnut_starch_NaOH, spoiled chestnut starch NaHSO_3_ + Bleach, and spoiled chestnut starch NaOH + Bleach.

### 2.14. Swelling Power, Starch Solubility, and Leached Amylose

The swelling behavior of chestnut starches was evaluated following the method of Srichuwong et al. [34]. Starch suspensions (1% *w*/*v*) were heated at 50, 60, 70, 80, and 90 °C for 30 min, with resuspension every 5 min. After heating, the tubes were rapidly cooled in tap water and centrifuged at 4500 rpm for 15 min. The supernatants were decanted, and the swollen starch precipitates were weighted.

The carbohydrate content of the supernatants was determined by the phenol–sulfuric acid method described by DuBois et al. [35] with slight modifications. Briefly, 10–150 µL of each supernatant was mixed with 200 µL of 5% (*w*/*v*) phenol solution in glass test tubes, followed by the addition of 1 mL of sulfuric acid (95–97% *v*/*v*) directly onto the mixture surface. After cooling, absorbance was measured at 490 nm using a GENESYS 50 UV-Visible spectrophotometer (Thermo Scientific, Waltham, MA, USA). The solubilized starch (SS) was expressed as the ratio of total carbohydrates in the supernatant to the starch dry matter. Swelling power was then calculated according to Equation (7):

(7) $$Swelling\;power\left(g\;water/g\;starch\right)=\frac{Weight\;of\;precipitate}{\begin{array}{c} starch\left(dry\;weight\right)\times \left(1-\frac{\%SS}{100}\right) \end{array}}$$

where SS is the soluble starch.

The amount of leached amylose (g/100 g starch dry weight) released during starch swelling was quantified by the colorimetric method of Chrastil [36], using maize starch with 66% amylose (Megazyme International Ireland Co., Ltd., Wicklow, Ireland) as the standard. Briefly, 2 mL of supernatant was transferred into a test tube containing 5 mL of 0.5% trichloroacetic acid and mixed. Afterwards, 0.05 mL of 0.01 N I2-KI solution (0.0127 g of I2 and 0.03 g of KI in 10 mL distilled H_2_O) was added. Samples were incubated at room temperature for 30 min, after which absorbance was recorded at 620 nm using a GENESYS 50 UV-Visible spectrophotometer (Thermo Scientific, Waltham, MA, USA). Swelling power, starch solubility, and leached amylose were determined in triplicate on 5 samples, namely the following: commercial corn starch, conforming_chestnut_starch_NaHSO_3_, conforming_chestnut_starch_NaOH, spoiled chestnut starch NaHSO_3_ + Bleach, and spoiled chestnut starch NaOH + Bleach.

### 2.15. Pasting Properties

The pasting properties of chestnut starch were evaluated using a starch pasting cell coupled to a controlled stress rheometer (AR-1000, TA Instruments, New Castle, DE, USA). Viscograms were obtained from 12% (*w*/*v*) starch–water suspensions, and viscosity data were processed with TA data analysis software (Version 5,0,8,0, TA Instruments, New Castle, DE, USA). Starch suspensions were stirred at 30 °C for 10 min under a constant shear rate of 60 s^−1^, which was maintained throughout the experiment. The temperature was then increased from 30 °C to 90 °C at a rate of 15 °C min^−1^ and held at 90 °C for 5 min, followed by cooling to 30 °C at the same rate and holding for 5 min. Commercial corn starch (Sigma, S-4126) was used as a reference.

For the comparison of the pasting properties among starches, the following parameters were determined from viscosity–temperature–time curves: gelatinization temperature (GT), defined as the temperature at which viscosity begins to increase; peak viscosity (PV), the maximum viscosity reached during heating; hot paste viscosity (HPV), the viscosity at the end of the isothermal period at 90 °C; cold paste viscosity (CPV), the viscosity at the end of the isothermal period at 30 °C; breakdown, calculated as PV–HPV; and setback, calculated as CPV–HPV. Pasting properties were evaluated in triplicate on 9 samples, namely the following: 12% (*m*/*v*) conforming_chestnut_starch_NaHSO_3_; 12% (*m*/*v*) conforming_chestnut_starch_NaOH; spoiled chestnut starch NaHSO_3_ + Bleach at 12%, 16%, and 18% (*m*/*v*); and spoiled chestnut starch NaOH + Bleach at 12%, 16%, 18%, and 20% (*m*/*v*).

### 2.16. Clarity Measurement (%T) on Starch Paste (Transmittance)

Starch paste clarity was evaluated by measuring light transmittance (%T) following the method described by Achille et al. [37], with modifications. Starch aqueous suspensions (12% *w*/*v*) were heated at 90 °C in a block heater for 30 min with resuspension every 5 min, then cooled to room temperature by immersion in tap water for 1 h. Light transmittance (%T) was measured at 650 nm against a water blank using a GENESYS 50 UV-Visible spectrophotometer (Thermo Scientific, Waltham, MA, USA). Clarity measurements were performed in triplicate on 6 samples, namely the following: commercial corn starch, potato starch, conforming_chestnut_starch_NaHSO_3_, conforming_chestnut_starch_NaOH, spoiled chestnut starch NaHSO_3_ + Bleach, and spoiled chestnut starch NaOH + Bleach.

### 2.17. Statistical Analysis

Statistical analysis was performed using GraphPad Prism 8 software (GraphPad Software, San Diego, CA, USA). Data were subject to an unpaired *t*-test or one-way analysis of variance (ANOVA) followed by Tukey’s post hoc multiple-comparison test. The level of significance used for all the statistical tests was 95% (*p* < 0.05).

## 3. Results and Discussion

### 3.1. Color Evaluation

High whiteness is a key quality attribute for starch used in the food industry. This is particularly true for products that require a clear appearance, such as batters, white sauces, and coatings. Therefore, the color of chestnut starches was measured and expressed in CIELab coordinates for straightforward comparison (Table 1). Representative images of chestnut flours and starch powders are shown in Figure 1.

To assess the effectiveness of the extraction methods in producing clear starches, the color of flours from conforming and spoiled chestnuts was evaluated. Flour from conforming chestnuts was light (L* = 89.55) and yellow (b* = 17.36; C* = 17.47; *p* < 0.0001), whereas flour from spoiled chestnuts was darker and less yellow (L* = 71.98; b* = 11.93; C* = 12.04; *p* < 0.0001).

When conforming chestnuts were extracted with either NaHSO_3_ or NaOH, the resulting native starches exhibited relatively low color saturation (C* = 10.91 and 13.81, respectively). This indicates the effective removal of yellow-pigmented compounds. In a previous study, Cruz et al. [16] reported a considerably lower color saturation (C* = 4.39) for starch from *C. sativa* Mill var. Longal, approximately 2.5- to 3-fold lower than the values obtained in the present study. However, Cruz et al. [16] performed starch extraction starting from milled, freeze-dried chestnuts. Following a similar approach, freeze-dried chestnuts were milled and steeped in NaHSO_3_ or NaOH solutions. This yield starches with comparable lightness but differing saturation values, C* = 6.66 for NaHSO_3_ and C* = 11.13 for the alkaline method. These results indicate that extraction from freeze-dried flour results in lower yellow pigmentation than extraction from whole kernels. These findings are consistent with Cruz et al. [16], who reported that NaHSO_3_ performs slightly better than alkaline treatment in producing clearer starch from freeze-dried chestnut flour.

The same extraction procedures, however, were ineffective in improving the color of starches isolated from spoiled chestnuts. To enhance whiteness, starch slurries were therefore treated with NaHSO_3_ or NaOH, supplemented with 33% (*v*/*v*) commercial sodium hypochlorite bleach (5% active chlorine). Hypochlorite treatment significantly increased lightness, with the highest value observed for the NaOH + Bleach combination (L* = 90.89), although color saturation (C*) remained largely unchanged. Despite this improvement, hypochlorite-treated chestnut starches, as well as all chestnut starches produced, exhibited similar lightness but retained a slight yellow hue perceptible to the naked eye (ΔE* = 19.45 for NaHSO_3_ + Bleach; ΔE* = 14.18 for NaOH + Bleach) compared to commercial corn starch. Although bleached starches may retain a light yellow hue, they remain highly functional for use in batters, sauces, coatings, and other food products where pure white color is not essential. Additionally, bleached starches can be utilized in unbleached paper production and have potential applications in biodegradable starch-based plastics.

### 3.2. Determination of Granule Structure and Size of Chestnut Starch by Scanning Electron Microscopy (SEM)

**Figure 2:**
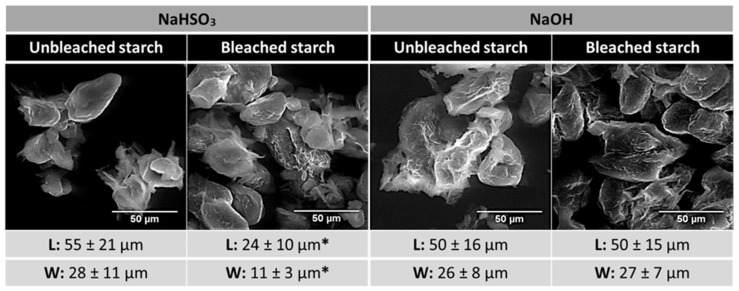
Representative micrographs of chestnut starches isolated from spoiled chestnuts using the NaHSO_3_, NaOH, NaHSO_3_ + Bleach, and NaOH + Bleach methods. The mean values and standard deviation (SD) of starch granules. * The dimensions shown correspond to the dispersed small-size granule fraction.

SEM was performed to evaluate the integrity of chestnut starch granules and the effect of the extraction methods on their structure. Starch granules isolated from spoiled chestnuts exhibited irregular morphologies, although elliptic- and rod-shaped granules were abundant (Figure 2). In addition, fractured surfaces were observed regardless of the steeping solution or the bleaching treatment. These anomalous structures are likely associated with microbial degradation occurring during spoilage, since starch granules extracted from conforming chestnuts, whether with NaHSO_3_ or NaOH, displayed smooth, intact surfaces without fractures [16,18].

Starches extracted with NaHSO_3_ solution had mean granule dimensions of 55 µm in length and 28 µm in width (Figure 2). In contrast, starches subjected to bleaching (NaHSO_3_ + Bleach) were smaller, with mean dimensions of 24 µm in length and 11 µm in width. However, accurate measurements of larger bleached granules were not possible due to poor dispersion, and thus, the reported values correspond only to the smaller granule fraction. For starches extracted using the alkaline method, bleaching did not significantly affect granule size. The mean length and width of NaOH- and NaOH + Bleach-treated starches were 50 µm and 26–27 µm, respectively, values comparable to those obtained for starches extracted with NaHSO_3_. Compared with previously reported values for native chestnut starch [16,18], the granules obtained from spoiled chestnuts were considerably larger.

### 3.3. Percentage of Humidity and Extraction Yield

To compare the moisture content of conforming and spoiled chestnuts, kernels were weighed before and after freeze-drying. Conforming kernels lost 47.4% of their mass, while spoiled kernels contained 44.1% water, a slightly lower value. These results suggest that microbial activity did not significantly affect water retention in spoiled kernels. This conclusion is further supported by the thermogravimetric analysis (TGA) of chestnut flours under 50% relative humidity, which showed no significant difference in water loss between conforming and spoiled samples (Table 2). When native and bleached chestnut starches were valuated under the same conditions, the mean water loss ranged from 8.8 to 10.6%, with no significant differences between them (*p* < 0.05).

Regarding extraction yield (Table 2), starch extracted using the NaHSO_3_ + Bleach method yielded 28.7%, which is lower than the 34.6% obtained with the NaOH + Bleach method. Overall, starch extraction from conforming chestnuts produced slightly higher yields compared to extraction from spoiled chestnuts.

A possible explanation for this difference is the lower starch content per unit mass in spoiled kernels, likely due to microbial degradation. Additionally, some starch may have been solubilized and lost during washing, as a result of oxidation and depolymerization induced by chlorine treatment [38]. Unquantified losses of starch during the multiple washing steps may also have contributed to the reduced total recovered material.

### 3.4. Aflatoxin Analysis

Aflatoxins are the only mycotoxins for which maximum regulatory limits have been established in tree nut food products. To assess the safety of bleached chestnut starches for human consumption, total aflatoxin contamination was evaluated in starches obtained by the NaHSO_3_ + Bleach and NaOH + Bleach treatments (Figures S1–S3, Supplementary Materials). In addition, spoiled chestnut flour was analyzed to determine whether spoiled chestnut kernels were contaminated with aflatoxins. The maximum permitted levels for aflatoxin B1 and total aflatoxins (B1, B2, G1, and G2) in tree nut products are 2 µg/kg and 4 µg/kg, respectively [39]. In all analyzed samples, total aflatoxin concentrations were below the method’s limit of detection (2 µg/kg). These results indicate that neither the bleached starches nor the original chestnut kernels contained aflatoxins at levels of toxicological concern.

### 3.5. Total Starch and Resistant Starch Content

Resistant starch is a dietary component that functions similarly to fiber, offering key benefits. It nourishes gut bacteria, reduces inflammation and colorectal cancer risk, regulates blood sugar by slowing glucose release, promotes satiety for weight control, and improves insulin sensitivity. These properties make it especially beneficial for individuals with diabetes or metabolic issues [10,11]. Considering its wide-ranging health benefits, resistant starch was investigated in bleached starches to explore its potential as a functional ingredient. This could enhance nutritional value while maintaining desirable food processing qualities.

**Figure 3:**
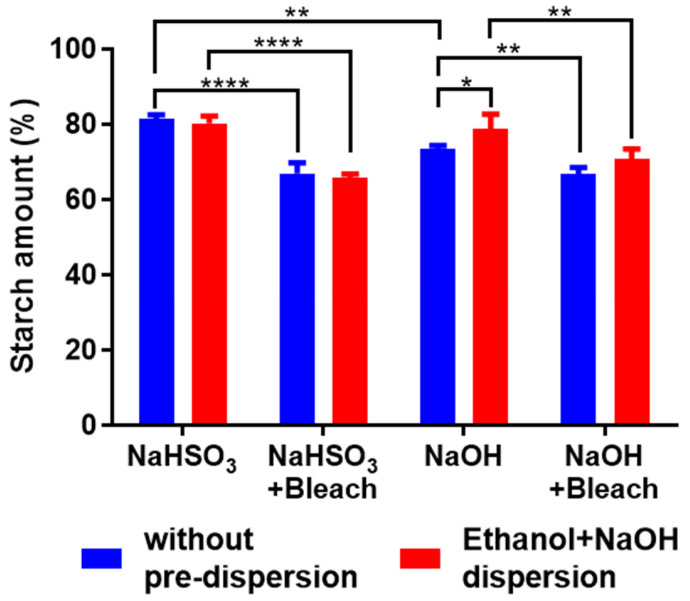
Total starch and soluble starch content. Blue bars represent soluble starch, while red bars represent total starch, which includes both soluble and insoluble fractions. Data were subject to one-way analysis of variance (ANOVA) followed by post hoc Tukey’s multiple-comparison test. Level of significance used for statistical tests was 95% (* *p* < 0.05; ** *p* < 0.01; **** *p* < 0.0001).

The hydrolysis patterns of native and bleached starches are shown in Figure 3. Since insoluble starch is resistant to α-amylase, enzymatic hydrolysis was performed both with and without pre-dispersion in ethanol and NaOH solutions (solubilization treatment). The solubilization treatment increased the hydrolysis percentage of NaOH and NaOH + Bleach starches by 5% and 4%, respectively. This reflects the proportion of insoluble and, therefore, resistant starch in these samples. In contrast, neither NaHSO_3_ nor NaHSO_3_ + Bleach starches contained insoluble starch.

The hydrolysis percentage of all samples varied between 66 and 81%, but the ending hydrolysis percentage of native starches was significantly higher (*p* < 0.0001) than those of bleached starches. For the NaHSO_3_ + Bleach group, the hydrolysis of starch showed a reduction around 14% (with and without solubilization treatment) compared with the native starch. In the NaOH group, with solubilization, the hydrolysis difference between native and bleached starches was 8%, which, combined with 4% insoluble starch, accounted for a total of 12% resistant starch.

These results indicate that the bleached starches do not contain sufficient resistant starch to be suitable for applications intended to replicate the functional properties of dietary fiber. However, the data do suggest that some degree of starch modification occurred, likely due to oxidation. This modification can interfere with the activity of α-amylase and/or amyloglucosidase. The production of resistant starch (11.9%) through oxidation was also reported by Zhou et al. [40], using 2.0 g active chlorine per 100 g of potato starch. A decrease in amyloglucosidase activity due to starch oxidation was similarly reported by Boruch [41].

### 3.6. Determination of Carboxyl and Carbonyl Group Contents in Chestnut Starches

Oxidation increases the polarity and hydrophilicity of starch through the conversion of hydroxyl groups at the C2, C3, and C6 positions of anhydroglucose units into carboxyl and carbonyl groups. Under harsh pH conditions, oxidation can also promote the hydrolysis of glycosidic bonds in amylose and amylopectin chains, leading to the formation of oligosaccharides. The rate of starch oxidation by sodium hypochlorite is strongly pH-dependent, since pH influences both the HOCl/ClO^−^ equilibrium in solution and the ionization state of starch. The pKa of hypochlorous acid (HOCl) is approximately 7.5; thus, at pH 8.5–10, chlorine exists mainly as ClO^−^, while at pH 4–6, HOCl predominates [42]. Under alkaline conditions, the hydroxyl groups of starch ionize, decreasing the oxidation rate due to repulsion between ClO^−^ anions and negatively charged starch chains [43]. Conversely, oxidation proceeds more rapidly near neutral pH, where ClO^−^ ions can react more effectively with neutral hydroxyl groups in starch [43,44].

Table 3 presents the percentage of carboxyl and carbonyl groups in bleached starches. The degree of oxidation was nearly 2-fold higher in starches obtained with the NaHSO_3_ + Bleach treatment (0.88%) compared with the NaOH + Bleach method (0.43%). The carbonyl content of NaHSO_3_ + Bleach starch was slightly higher (*p* < 0.05) compared to NaOH + Bleach, while the carboxyl content was 4-fold greater (*p* < 0.01). These differences can be attributed to variations in the pH of the steeping solutions.

In the NaOH + Bleach treatment, the pH decreased from 12.7 at the start of the reaction to 9.6 after 24 h. Although ClO^−^ concentration is maximal within this pH range, the deprotonation of starch hydroxyl groups imparts negative charges to the chains, which hinders oxidation [43]. In addition, part of ClO^−^ may have been consumed during the oxidation of the degraded tissues of spoiled chestnut kernel, reducing the availability of the oxidant for amylose and amylopectin. This interpretation is supported by the higher lightness value observed for NaOH + Bleach starch (L* = 90.89), compared with starch treated at lower pH (L* = 83.82; Table 3).

In contrast, the NaHSO_3_ + Bleach treatment was carried out at pH 6.2–8.5. This is the range in which sodium hypochlorite exhibits its maximum starch oxidation activity [42,43]. As a result, the starch obtained had a higher degree of oxidation.

### 3.7. Characterization of Bleached Starches Using Electrospray Ionization–Mass Spectrometry (ESI-MS)

**Figure 4:**
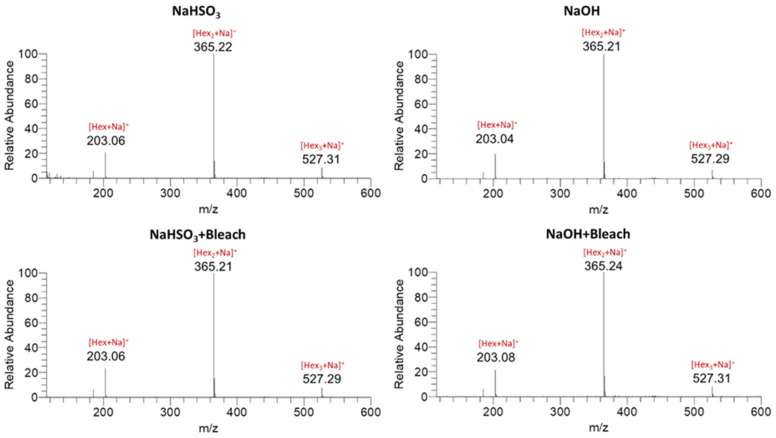
ESI-MS spectra of native and bleach-treated chestnut starches extracted with NaHSO_3_, NaOH, NaHSO_3_ + Bleach, and NaOH + Bleach.

In Figure 4, the ESI-MS spectra of native and bleach-treated chestnut starches are presented. For the native starch, ions were detected at *m*/*z* 203, 365, and 527, corresponding to the sodium adducts of hexose ([Hex + Na]^+^), maltose ([Hex2 + Na]^+^), and maltotriose ([Hex3 + Na]^+^), respectively. The spectra of the bleach-treated starches showed the same ions (*m*/*z* 203, 365, and 527) and showed no additional signals. This indicates that the bleach-treated starches did not contain significant detectable levels of oxidized hydroxyl groups, such as carbonyl or carboxyl moieties. These findings are consistent with the titration results (Table 3), confirming that the bleached starches exhibited only a low degree of oxidation.

### 3.8. Characterization of Bleached Starches Using High-Performance Size Exclusion Chromatography (HPSEC)

**Figure 5:**
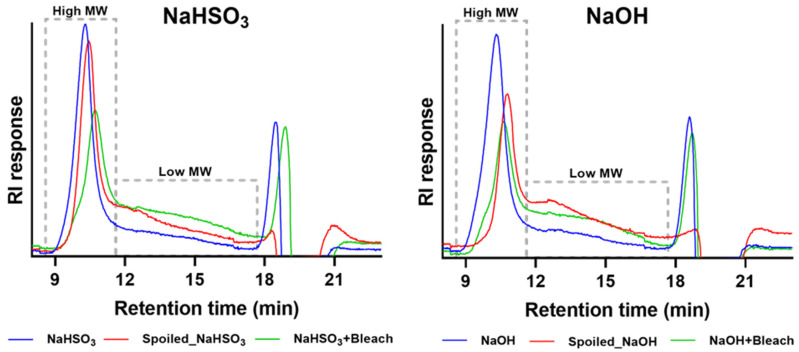
High-performance size exclusion chromatograms of starches extracted from conforming chestnut (NaHSO_3_ and NaOH), spoiled chestnut starches (Spoiled_chestnut_starch_NaHSO_3_ and Spoiled_chestnut_starch_NaOH), and bleached (spoiled) chestnut starches (NaHSO_3_ + Bleach and NaOH + Bleach). Chromatograms represent the normalized refractive index (RI) response. High MW = high-molecular-weight fraction; Low MW = low-molecular-weight fraction.

The molecular weight (MW) profiles of bleached chestnut starches obtained from spoiled kernels (NaHSO_3_ + Bleach and NaOH + Bleach) were determined by high-performance size exclusion chromatography. These were compared with starches from conforming chestnut (NaHSO_3_ and NaOH) and non-bleached starches from spoiled kernels (Spoiled_chestnut_starch_NaHSO_3_ and Spoiled_chestnut_starch_NaOH). Figure 5 shows the distribution of high- and low-MW fractions of starches extracted in the presence of either NaHSO_3_ or NaOH. The high-MW fractions of all starches eluted between 10.4 and 10.8 min, with no significant differences among the different extraction methods.

On the other hand, the proportions of the high- and low-MW fractions, corresponding mainly to amylopectin and amylose, respectively [26], varied more noticeably among starch samples. The percentages of the low-MW fraction (eluting after 10.8 min) of NaHSO_3_, Spoiled_chestnut_starch_NaHSO_3_, and NaHSO_3_ + Bleach were 29 ± 5%, 38 ± 4%, and 46 ± 2%, respectively, with significant difference between NaHSO_3_ and NaHSO_3_ + Bleach (*p* < 0.01). This increase in the low-MW fraction in NaHSO_3_ + Bleach starch likely reflects the partial degradation of amylopectin, as both amylopectin and amylose chains are known to undergo oxidative cleavage [25]. For NaOH-extracted starch from conforming chestnut, the low-MW fraction of 23 ± 3% was significantly lower (*p* < 0.0001) than those of Spoiled_chestnut_starch_NaOH (46 ± 2%) and NaOH + Bleach (47 ± 1%), suggesting that alkaline extraction alone was sufficient to promote amylopectin erosion in starch from spoiled chestnut.

Overall, the effect of bleaching on starch MW profiles was inconclusive. Both spoiled chestnut starches (regardless of bleaching) showed higher proportions of low-MW fractions compared with starches obtained from conforming kernels.

### 3.9. Thermogravimetric Analysis (TGA)

**Figure 6:**
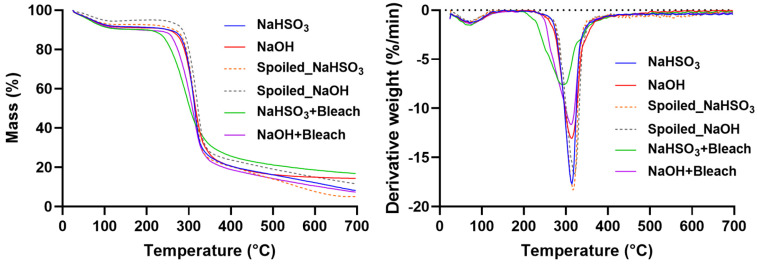
Thermogravimetric (TGA, left) and derivative thermogravimetric (DTG, right) curves of native and oxidized chestnut starches extracted with NaHSO_3_, NaOH, NaHSO_3_ + Bleach, and NaOH + Bleach.

The thermal decomposition profiles of starch provide insights into differences in starch composition, such as the amylose-to-amylopectin ratio [45], as well as chemical modifications that affect gelatinization properties [26,27,46]. To assess these characteristics, thermogravimetric analysis (TGA) was performed on native and bleached starches. Figure 6 presents the weight loss and derivative thermogravimetry (DTG) curves for the samples, while Table 4 summarizes the onset temperatures and the temperatures corresponding to the maximum rate of weight loss.

The TGA curves revealed a two-step weight loss for all samples. The first, ranging from 7.9% to 10.6% of the starch mass, occurred near 74 °C and corresponds to the loss of adsorbed water molecules. The second, larger weight loss is associated with the decomposition of amylose and amylopectin into CO, CO_2_, H_2_O, CH_4_, C_2_H_4_, and CH_2_O [45]. For NaHSO_3_, NaOH, and NaOH + Bleach starches, the maximum decomposition occurred around 314 °C, whereas for NaHSO_3_ + Bleach it occurred at a lower temperature of approximately 297 °C (Table 4). Starch extracted from spoiled chestnuts using NaHSO_3_ or NaOH, without a bleaching step, exhibited higher maximum decomposition temperatures than the native starches, reaching 317 °C and 320 °C, respectively.

The onset temperature of NaOH + Bleach was 8 °C lower than that of its native counterparts, while NaHSO_3_ + Bleach exhibited a 36.7 °C lower onset than native NaHSO_3_ starch (*p* < 0.0001), indicating significantly reduced thermal stability (*p* < 0.001) relative to NaOH + Bleach. This difference cannot be attributed to the spoilage-induced degradation of the raw material, as the onset decomposition temperatures are comparable to those of the native starches. These results are consistent with previous studies showing that oxidation decreases starch thermostability [21,27]. The oxidation of hydroxyl groups to carbonyl at the C2, C3, and C6 positions weakens the hydrogen bonds stabilizing the starch structure [23], lowering the decomposition temperature. Since NaHSO_3_ + Bleach starch exhibited approximately twice the oxidation degree of NaOH + Bleach (Table 3), its thermal destabilization is more pronounced, with a decrease of 17 °C relative to the respective control. In contrast, the peak decomposition temperatures of NaOH + Bleach starch and conforming NaOH starch did not show a significant variation. Extensive oxidation at the C2 or C3 position may also promote the depolymerization of starch chains via β-elimination, reducing molecular weight [23,45]. Despite the reduced thermal stability of bleached starches, their decomposition temperatures remain well above typical food processing temperatures (<255 °C), allowing for their safe use in food products.

### 3.10. Crystal Conformation (XRD)

To evaluate the effect of the extraction methods on the long-range crystalline structure of starches, powder X-ray diffraction (XRD) analysis was performed. Conforming and spoiled chestnut flours were used as references.

**Figure 7:**
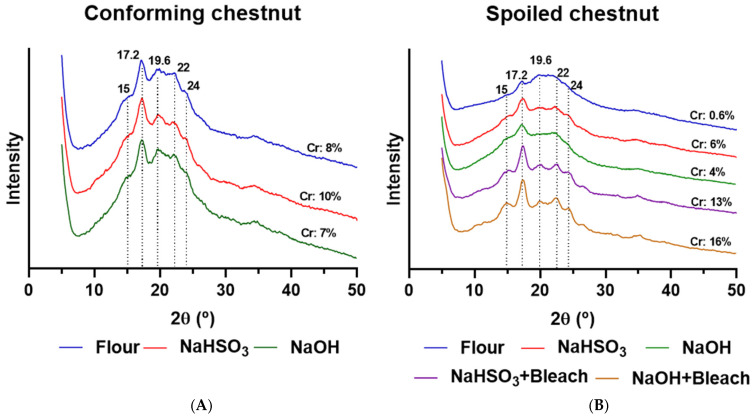
X-ray diffraction patterns and degree of crystallinity (%Cr) of chestnut starches extracted with NaHSO_3_, NaOH, NaHSO_3_ + Bleach, and NaOH + Bleach from conforming (**A**) and spoiled (**B**) chestnuts.

The X-ray diffractograms of chestnut flours and starches are shown in Figure 7. The isolation of native chestnut starch (Figure 7A) using either NaHSO_3_ or NaOH resulted in a strong peak at 2θ near 17°, a shoulder peak at 15°, and minor peaks at 19.6°, 22°, and 24°, consistent with a CB-type conformation [14]. The degree of crystallinity (%Cr) was 10% for NaHSO_3_ extracted starch and 7% for NaOH extracted starch. These results agree with those of Correia et al. [14], who also observed a CB-type diffraction pattern in chestnut starch from the Longal and Martainha cultivars, although their samples showed more intense peaks and higher crystallinity (34.8–36%). In contrast, Cruz et al. [16] observed a B-type pattern in Longal starch extracted with NaHSO_3_. Regarding %Cr, both Cruz et al. [16] and Bogracheva et al. [47] demonstrated that starch crystallinity is strongly influenced by hydration level, suggesting that differences in water content under the experimental conditions may explain the lower relative crystallinity observed.

Interestingly, conforming chestnut flour exhibited the same XRD pattern as native starch (Cr = 8%) despite not undergoing any purification treatment (Figure 7A). In contrast, spoiled chestnut flour displayed an amorphous structure (Figure 7B). Starches extracted from spoiled kernels using NaHSO_3_ or NaOH yielded starches with 6% and 4% crystallinity, respectively, characterized by strong peaks at 17°, shoulder peaks at 15°, and minor peaks near 22° (Figure 7B). The degree of crystallinity increased significantly when extraction was combined with bleaching, reaching 13% and 16% for NaHSO_3_ + Bleach and NaOH + Bleach starches, respectively. In both cases, the XRD patterns showed peaks at 15°, 17°, 19.6°, 22°, and 24°, consistent with the CB-type conformation observed in native starches. This increase in crystallinity following bleaching may be attributed to oxidation and removal of molecules attached to starch granules. The preferential erosion of amorphous regions enhances the relative proportion of crystalline domains [25,26].

### 3.11. Chestnut Starch Crystallinity Assessed by ATR-FTIR

**Figure 8:**
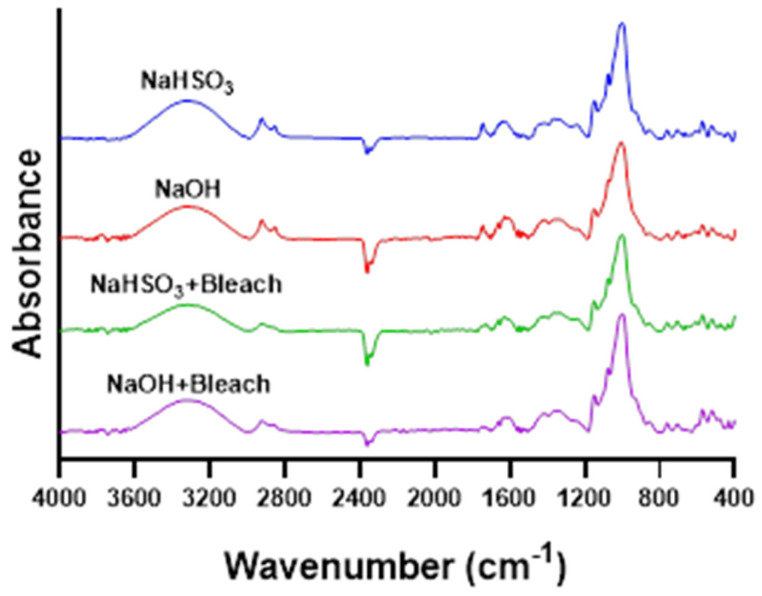
ATR-FTIR spectra of native (NaHSO_3_ and NaOH) and bleached (NaHSO_3_ + Bleach and NaOH + Bleach) chestnut starches.

The FTIR spectrum of starch is sensitive to changes in short-range molecular order. This includes alterations in chain conformation, crystallinity, retrogradation, and water content [48]. The region between 1300 and 800 cm^−1^ is characteristic for starch (Figure 8). Within this region, the IR absorbance bands at 1022 cm^−1^ and 1047 cm^−1^ are associated with amorphous and crystalline structures, respectively. The 1047/1022 cm^−1^ ratio is commonly used to quantify the proportion of ordered crystalline domains relative to amorphous regions in starch [48,49,50].

The band at 992 cm^−1^, sensitive to starch hydration state, overlaps with and influences the position of the 1022 cm^−1^ band [48]. In hydrated chestnut starches, this 992 cm^−1^ band shifts toward around 1000 cm^−1^, causing the 1022 cm^−1^ band to shift to approximately 1013 cm^−1^. The band previously reported at 1047 cm^−1^ was more accurately reassigned to 1040 cm^−1^. Consequently, the 1040/1013 cm^−1^ ratio was used in place of the 1047/1022 cm^−1^ ratio.

Native starch isolated with NaOH exhibited the highest 1040/1013 ratio (0.83), indicating the greatest short-range ordering of starch chains among the samples (Table 5). NaHSO_3_ + Bleach starch had the second highest ratio (0.76), while NaHSO_3_ and NaOH + Bleach starches showed slightly lower values of 0.73 and 0.74, respectively. Overall, none of the samples displayed low 1040/1013 ratios, suggesting that all starches maintained a substantial short-range molecular order and crystalline structure. Based on these results, no clear relationship between extraction method and crystallinity could be established.

### 3.12. Swelling Power, Starch Solubility, and Leached Amylose

**Figure 9:**
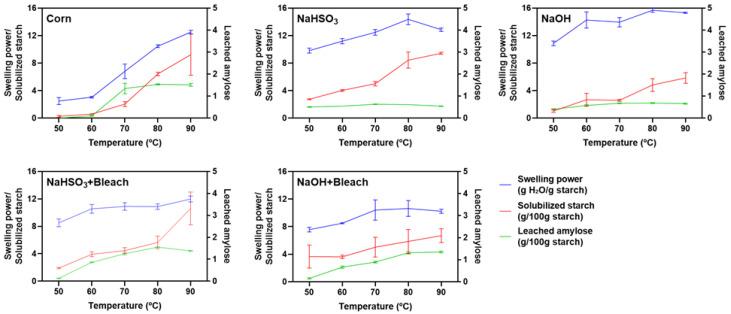
Swelling power (g H_2_O/g starch), solubilized starch (g/100 g starch), and leached amylose (g/100 g starch) of chestnut starches extracted from conforming kernels with NaHSO_3_ or NaOH and from spoiled kernels with NaHSO_3_ + Bleach or NaOH + Bleach. Commercial corn starch was included as reference.

The swelling of starch granules occurs when granules are heated in excess water, and swelling power is a key property influencing pasting behavior and rheological characteristics. The textural properties of starchy foods are also affected by the extent of starch solubilization and amylose leaching. Therefore, swelling power, solubilized starch, and leached amylose were evaluated between 50 °C and 90 °C (Table 6, Table 7 and Table 8, respectively). Temperature had a significant effect on all parameters (*p* < 0.0001), with values increasing progressively as temperature rose (Figure 9).

Corn starch, used as a reference, exhibited a continuous increase in swelling power, reaching its maximum at 90 °C. Remarkably, all chestnut starches showed significantly higher swelling power than corn starch between 50 and 70 °C (*p* < 0.01). Native chestnut starches isolated with either NaHSO_3_ or NaOH also maintained higher swelling power than corn starch up to 80 °C (*p* < 0.001). Between 70 and 90 °C, the swelling power of bleached chestnut starches remained nearly constant. At 90 °C, however, corn starch displayed significantly higher swelling power than both NaHSO_3_ + Bleach and NaOH + Bleach starches (*p* < 0.001).

Overall, bleached starches exhibited significantly lower swelling power than their corresponding native counterparts (*p* < 0.05; Table 6). This reduction cannot be explained by starch solubilization on its own (Table 7), as significant differences were only observed between NaOH and NaOH + Bleach at 50 °C and 70 °C (*p* < 0.01). Both starch solubilization and amylose leaching (Figure 9 and Table 8) correlated positively with swelling power up to 80 °C across all samples. However, at 90 °C, these trends reversed (Figure 9). At 90 °C, the excessive solubilization of amylopectin and amylose leaching compromise granule integrity, reducing water absorption capacity. A similar phenomenon was reported by Srichuwong et al. [34].

Regarding the effect of extraction method on native starches, starch isolated under alkaline conditions displayed higher swelling power than that obtained with NaHSO_3_ (Table 6). The greatest differences occurred at 60 °C (*p* < 0.01) and 90 °C (*p* < 0.0001). In contrast, differences between bleached starches were less marked. NaHSO_3_ + Bleach exhibited significantly higher swelling power than NaOH + Bleach only at 60 °C and 90 °C (*p* < 0.0001).

### 3.13. Pasting Properties

Profiling the pasting properties of starch is an effective approach to establishing correlations between starch functionality and structural characteristics. Such analysis provides valuable insights into potential industrial applications, particularly for products that rely on starch viscosity and thickening behavior.

**Figure 10:**
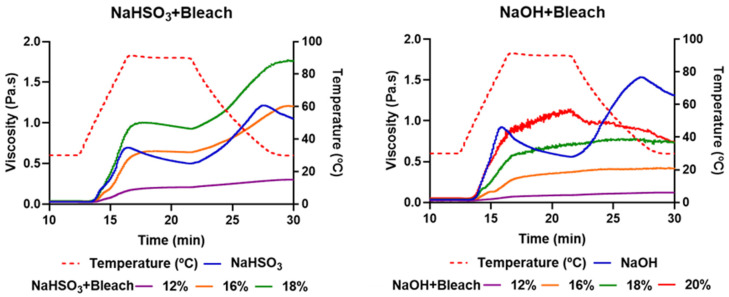
Viscograms of chestnut starches isolated using the NaHSO_3_, NaOH, NaHSO_3_ + Bleach, and NaOH + Bleach methods. Bleached starches were tested at 12%, 16%, 18%, and 20% (*m*/*v*). The first 10 min of each run were omitted, as this period corresponds to starch resuspension and homogenization.

The pasting properties of corn and chestnut starches isolated with NaHSO_3_ or NaOH and dispersed in water at a concentration of 12% (*m*/*v*) are summarized in Table 9. Viscograms (Figure 10) were used to calculate gelatinization temperature (GT), peak viscosity (PV), hot paste viscosity (HPV), cold paste viscosity (CPV), breakdown (PV-HPV), and setback (CPV-HPV). These parameters allow for a comparison of pasting behavior across the different starch samples.

Distinct viscosity profiles were observed depending on the extraction method applied. Consistent with the swelling power results, chestnut starches exhibited lower GT values (ranging 50–54.3 °C) compared to corn starch (67.6 °C), reflecting a less rigid starch structure and requiring lower energy for gelatinization. Extraction from conforming kernels with either NaHSO_3_ or NaOH did not significantly affect GT, and only minor variations were observed in bleached starches as starch concentration increased.

Corn starch exhibited a lower PV (0.468 Pa s^−1^) compared to conforming chestnut starch isolated with NaHSO_3_ (0.696 Pa s^−1^) or NaOH (0.899 Pa s^−1^), reflecting the greater swelling power of native chestnut starches.

In contrast, bleached chestnut starches showed the lowest PV values, consistent with previous reports on oxidized potato, corn, rice, and barley starches [25,26]. At a concentration of 12% (*m*/*v*), oxidized starches generally exhibited low viscosity, likely due to the structural loosening or partial breakdown of the starch granules, which facilitates water penetration. Interestingly, NaHSO_3_ + Bleach starch regained gelatinization capacity at 18% (*m*/*v*), as reflected by a broader PV (1.03 Pa s^−1^) (Figure 10). In contrast, the viscosity profile of NaOH + Bleach (Figure 10) shows that when exposed to heat and shear stress, the starch granules rapidly lose structural integrity, preventing the development of a viscosity peak [26]. Due to polysaccharide degradation, the starch chains are unable to re-associate during retrogradation [25], and the modest increase in viscosity reflects only the effect of cooling.

Chestnut starch isolated with NaHSO_3_ exhibited a breakdown of 0.193, comparable to that of corn starch (0.183), indicating moderate heat stability. In contrast, starch isolated using NaOH displayed a significantly higher breakdown (0.336; *p* < 0.001), reflecting reduced thermal stability likely caused by structural weakening. Notably, NaHSO_3_ + Bleach starch at 18% (*m*/*v*) exhibited the lowest breakdown (0.10), indicative of strong heat stability. Conversely, the negative breakdown values observed under the remaining conditions (Table 9) confirmed the inability of these starch dispersions to form gels.

Corn starch showed moderate retrogradation (0.371), which was significantly lower (*p* < 0.01) than that of NaOH starch (0.744). NaHSO_3_ chestnut starch had an intermediate setback value (0.530), suggesting that native chestnut starches undergo stronger gelatinization due to amylose retrogradation compared to corn starch. The setback of NaHSO_3_ + Bleach starch increased with concentration, reaching as high as 0.821 at 18% (*m*/*v*). In contrast, the setback values of NaOH + Bleach starch decreased as starch concentration increased (Table 9), indicating reduced retrogradation. This reduction may be attributed to amylose degradation and the presence of carbonyl and carboxyl groups, which disrupt amylose re-association and hinder gel formation [25].

Overall, native chestnut starches exhibit lower gelatinization temperatures and higher peak viscosities compared to corn starch. Among the native chestnut starches, the alkaline-isolated sample produced pastes with the highest breakdown and setback viscosities, indicating moderate thermal stability but enhanced retrogradation. NaHSO_3_ + Bleach starch required a higher concentration than the native starch to achieve a comparable viscosity profile. In contrast, NaOH + Bleach starch lost both gelatinization and retrogradation properties.

### 3.14. Clarity Measurement of Starch Pastes (Transmittance, %T)

**Figure 11:**
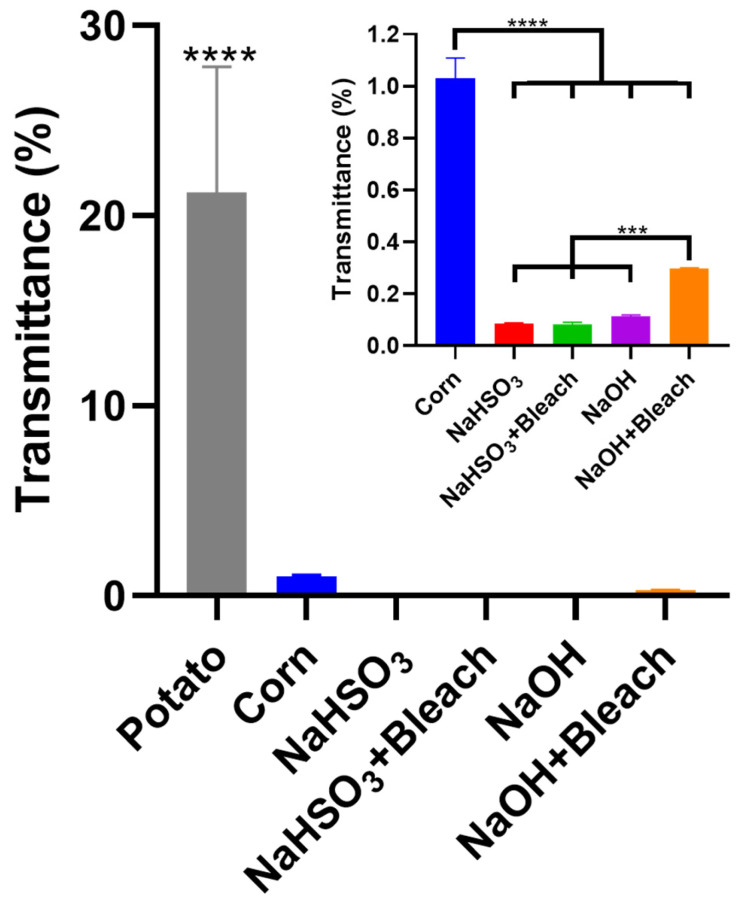
Transmittance (%) of chestnut starch pastes extracted using NaHSO_3_, NaOH, NaHSO_3_ + Bleach, and NaOH + Bleach methods. Commercial potato and corn starches were used as reference samples. All starches were tested at 12% (*m*/*v*). Data were subject to one-way analysis of variance (ANOVA) followed by Tukey’s post hoc multiple-comparison test. Level of significance used for statistical tests was 95% (*** *p* < 0.001; **** *p* < 0.0001).

The clarity of starch pastes is an important property in many food applications. For instance, gelatinized starch must feature high transparency to incorporate jams and gelatins [21,22]. Hence, light transmittance through gelatinized chestnut starches was evaluated, as shown in Figure 11, with potato and corn starches as reference samples. Potato starch produced pastes with an average light transmittance of 21%, which was significantly higher (*p* < 0.0001) than that of all other samples. In contrast, corn starch paste exhibited much lower clarity, with a transmittance of just 1%T, yet it was still significantly (*p* < 0.001) clearer than the chestnut starch pastes. Among the native chestnut starch samples, the NaOH starch paste showed a slightly higher transmittance (0.114%T) than the NaHSO_3_ counterparts, although this difference was not statistically significant. The opaquest pastes were produced by the NaHSO_3_ and NaHSO_3_ + Bleach treatments, with transmittance values of 0.087%T and 0.084%T, respectively. Of all the chestnut starch samples, the NaOH + Bleach starch paste exhibited the highest clarity, with a transmittance of 0.297%T. However, the viscosity profile (Figure 10) indicates that no retrogradation took place. Therefore, the increased transmittance is likely due to structural differences in the NaOH + Bleach paste compared to the other chestnut starch pastes.

## 4. Conclusions

Bleach-treated chestnut starch derived from spoiled kernels presents several functional properties that make it suitable for industrial applications. Owing to its moderate gelatinization properties and high swelling capacity at low temperatures, it can be effectively used as a thickening agent in batters, sauces, coatings, and deserts requiring a firm texture. Additionally, bleached chestnut starches can be utilized in unbleached paper formulations and have potential applications in biodegradable starch-based plastics.

The bleach treatment of starch isolated from spoiled chestnuts increased lightness, although some residual yellow pigmentation remained. Spoiled chestnut starch granules exhibited fractured surfaces regardless of steeping or bleaching treatment. Notably, aflatoxin was not detected. Starch granules also contained a higher proportion of low-molecular-weight carbohydrates, likely due to microbial degradation during spoilage. Despite this, bleached starches showed an improved degree of crystallinity compared to native chestnut starch. Among the bleached starches, NaHSO_3_ + Bleach starch exhibited approximately twice the degree of oxidation compared to NaOH + Bleach starch, resulting in more pronounced thermal destabilization.

Chestnut starches demonstrated higher swelling power at temperatures below 90 °C and lower gelatinization temperatures with higher peak viscosities compared to corn starch. While NaHSO_3_ + Bleach starch required higher concentrations to achieve viscosities comparable to native starch, the viscosity of NaOH + Bleach starch exhibited only a minimal increase. Corn starch pastes were significantly clearer than those formed by chestnut starches.

Based on these results, oxidative extraction at middle pH proved to be the most effective method for recovering functional starch from spoiled chestnuts. Subsequent research will assess the levels of chemical and microbiological impurities within bleached starch. To further expand its industrial value beyond the food sector, bleached chestnut starches will be evaluated for use in unbleached paper production and in the development of biodegradable starch-based films.

## Figures and Tables

**Table 2:** Percentage of moisture in chestnut flours, and chestnut starches obtained using sodium bisulfite (NaHSO_3_) or alkaline (NaOH) extraction methods determined using thermogravimetric analysis (TGA).

Samples	Moisture (%)	Extraction Yield (%)
Conforming chestnut flour	8.2	n.a.
Spoiled chestnut flour	9.1	n.a.
Conforming_chestnut_starch_NaHSO_3_	10.6	40.6
Conforming_chestnut_starch_NaOH	9.0	35.8
Spoiled_chestnut_starch_NaHSO_3_ + Bleach	8.8	28.7
Spoiled_chestnut_starch_NaOH + Bleach	9.8	34.6

n.a.—not applicable.

**Table 3:** Carboxyl and carbonyl group contents of bleached starches.

Extraction Method	pH t = 0 h	pH t = 24 h	Carboxyl Groups (%)	Carbonyl Groups (%)	Total Oxidation (%)
NaHSO_3_ + Bleach	8.5	6.2	0.38 ± 0.02 ^a^	0.50 ± 0.06 ^a^	0.88
NaOH + Bleach	12.7	9.6	0.09 ± 0.06 ^b^	0.34 ± 0.06 ^b^	0.43

Values are expressed as means of triplicates ± standard deviations. Data were subject to unpaired *t*-test. Means within same column followed by different superscript letters differ significantly (*p* < 0.05).

**Table 4:** Thermogravimetric analysis of native and oxidized starches.

Extraction Method	Peak Temperature (°C)	Onset (°C)
NaHSO_3_ (conforming chestnut)	313.8 ± 0.9 ^a^	291.3 ± 0.5 ^a^
NaOH (conforming chestnut)	313.4 ± 0.9 ^a^	286.7 ± 1.7 ^a,b^
Spoiled NaHSO_3_ (spoiled chestnut)	317.0 ± 1.7 ^a^	288.5 ± 3.6 ^a^
Spoiled NaOH (spoiled chestnut)	319.6 ± 1.1 ^a^	287.2 ± 2.2 ^a,b^
NaHSO_3_ + Bleach (spoiled chestnut)	296.8 ± 4.2 ^b^	254.6 ± 4.2 ^c^
NaOH + Bleach (spoiled chestnut)	315.6 ± 3.2 ^a^	278.5 ± 5.1 ^b^

Values are expressed as means of triplicates ± standard deviations. Data were subject to one-way analysis of variance (ANOVA) followed by Tukey’s post hoc multiple-comparison test. Means within same column followed by different superscript letters differ significantly (*p* < 0.05).

**Table 5:** Ratios of infrared absorbance bands at 1040 and 1013 cm^−1^ in starch samples.

Extraction Method	R(1040/1013)
NaHSO_3_ (conforming chestnut)	0.73
NaOH (conforming chestnut)	0.83
NaHSO_3_ + Bleach (spoiled chestnut)	0.76
NaOH + Bleach (spoiled chestnut)	0.74

**Table 6:** Swelling power (g H_2_O/g starch) of chestnut starches extracted from conforming kernels using NaHSO_3_ or NaOH and from spoiled kernels using NaHSO_3_ + Bleach or NaOH + Bleach. Commercial corn starch was included as reference.

Swelling Power (g H_2_O/g Starch)	Temperature (°C)				
Starch Samples	50	60	70	80	90
Corn	2.49 ± 0.50 ^a^	3.05 ± 0.13 ^a^	6.81 ± 1.05 ^a^	10.46 ± 0.20 ^a^	12.51 ± 0.26 ^b,c^
NaHSO_3_	9.82 ± 0.36 ^c^	11.19 ± 0.39 ^c^	12.47 ± 0.41 ^b,c^	14.37 ± 0.79 ^b^	12.86 ± 0.26 ^c^
NaOH	10.88 ± 0.34 ^c^	14.27 ± 1.17 ^d^	13.96 ± 0.68 ^c^	15.68 ± 0.28 ^b^	15.33 ± 0.10 ^d^
NaHSO_3_ + Bleach	8.51 ± 0.56 ^b,c^	10.55 ± 0.65 ^c^	10.91 ± 0.56 ^b,c^	10.88 ± 0.40 ^a^	11.98 ± 0.43 ^b^
NaOH + Bleach	7.57 ± 0.35 ^b^	8.50 ± 0.09 ^b^	10.41 ± 1.48 ^b^	10.63 ± 1.16 ^a^	10.23 ± 0.30 ^a^

Values are expressed as means of triplicates ± standard deviations. Data were subject to one-way analysis of variance (ANOVA) followed by Tukey’s post hoc multiple-comparison test. Means within the same column followed by different superscript letters differ significantly (*p* < 0.05).

**Table 7:** Solubilized starch (g/100 g starch) in chestnut starches extracted from conforming kernels using NaHSO_3_ or NaOH and from spoiled kernels using NaHSO_3_ + Bleach or NaOH + Bleach. Commercial corn starch was included as reference.

Solubilized Starch (g/100 g Starch)	Temperature (°C)				
Starch Samples	50	60	70	80	90
Corn	0.26 ± 0.14 ^a^	0.60 ± 0.03 ^a^	2.04 ± 0.36 ^a^	6.43 ± 0.25 ^a,b^	9.23 ± 2.99 ^a,b^
NaHSO_3_	2.74 ± 0.07 ^c^	4.04 ± 0.14 ^c^	5.01 ± 0.32 ^b^	8.43 ± 1.17 ^b^	9.44 ± 0.15 ^a,b^
NaOH	1.05 ± 0.21 ^b^	2.67 ± 0.94 ^b^	2.60 ± 0.14 ^a^	4.82 ± 0.92 ^a^	5.85 ± 0.77 ^a^
NaHSO_3_ + Bleach	1.91 ± 0.10 ^b,c^	3.93 ± 0.35 ^b,c^	4.46 ± 0.43 ^b^	5.65 ± 0.93 ^a,b^	10.60 ± 2.39 ^b^
NaOH + Bleach	3.67 ± 1.67 ^c^	3.62 ± 0.26 ^b,c^	5.02 ± 1.44 ^b^	5.85 ± 1.74 ^a,b^	6.70 ± 1.00 ^a,b^

Values are expressed as means of triplicates ± standard deviations. Data were subject to one-way analysis of variance (ANOVA) followed by Tukey’s post hoc multiple-comparison test. Means within same column followed by different superscript letters differ significantly (*p* < 0.05).

**Table 8:** Leached amylose (g/100 g starch) in chestnut starches extracted from conforming kernels using NaHSO_3_ or NaOH and from spoiled kernels using NaHSO_3_ + Bleach or NaOH + Bleach. Commercial corn starch was included as reference.

Leached Amylose (g/100 g Starch)	Temperature (°C)				
Starch Samples	50	60	70	80	90
Corn	ND	0.10 ± 0.03 ^a^	1.35 ± 0.24 ^c^	1.54 ± 0.03 ^d^	1.51 ± 0.07 ^d^
NaHSO_3_	0.51 ± 0.02 ^b^	0.545 ± 0.003 ^b^	0.63 ± 0.01 ^a^	0.62 ± 0.00 ^a^	0.54 ± 0.01 ^a^
NaOH	0.40 ± 0.03 ^b^	0.58 ± 0.04 ^b^	0.68 ± 0.02 ^a^	0.69 ± 0.02 ^b^	0.66 ± 0.03 ^b^
NaHSO_3_ + Bleach	0.13 ± 0.02 ^a^	0.86 ± 0.02 ^c^	1.25 ± 0.06 ^c^	1.55 ± 0.01 ^d^	1.38 ± 0.02 ^c^
NaOH + Bleach	0.15 ± 0.03 ^a^	0.67 ± 0.05 ^b,c^	0.90 ± 0.04 ^b^	1.31 ± 0.05 ^c^	1.35 ± 0.04 ^c^

Values are expressed as means of triplicates ± standard deviations. Data were subject to one-way analysis of variance (ANOVA) followed by Tukey’s post hoc multiple-comparison test. Means within same column followed by different superscript letters differ significantly (*p* < 0.05). ND—Non-detectable.

**Table 9:** Pasting properties of chestnut starches extracted using NaHSO_3_, NaOH NaHSO_3_ + Bleach, and NaOH + Bleach methods. Commercial corn starch was used as reference. Starches were tested at concentration of 12% (*m*/*v*), while bleached starches were additionally tested at 16%, 18%, and 20% (*m*/*v*).

Starch Samples	Concentration (*m*/*v*)	Gelatinization Temperature (GT) (°C)	Peak Viscosity (PV) (Pa s^−1^)	Hot Paste Viscosity (HPV) (Pa s^−1^)	Cold Paste Viscosity (CPV) (Pa s^−1^)	Breakdown (PV–HPV)	Setback (CPV–HPV)
Corn	12%	67.6 ± 0.2 ^d^	0.468 ± 0.002 ^b^	0.285 ± 0.001 ^b^	0.655 ± 0.009 ^b^	0.183 ± 0.001 ^c^	0.371 ± 0.009 ^b^
NaHSO_3_	12%	50.5 ± 0.17 ^a^	0.696 ± 0.001 ^c^	0.503 ± 0.011 ^c^	1.034 ± 0.014 ^c^	0.193 ± 0.012 ^c^	0.530 ± 0.025 ^b^
NaHSO_3_ + Bleach	12%	52.7 ± 0.6 ^b^	0.173 ± 0.006 ^a^	0.207 ± 0.013 ^b^	0.300 ± 0.020 ^a^	−0.034 ± 0.007 ^b^	0.092 ± 0.007 ^b^
NaHSO_3_ + Bleach	16%	54.3 ± 0.7 ^c^	0.574 ± 0.067 ^b^	0.639 ± 0.048 ^c^	1.198 ± 0.188 ^c^	−0.065 ± 0.096 ^b^	0.559 ± 0.194 ^b^
NaHSO_3_ + Bleach	18%	53.9 ± 1.2 ^c^	1.031 ± 0.057 ^e^	0.928 ± 0.146 ^e^	1.749 ± 0.129 ^d^	0.103 ± 0.092 ^c^	0.821 ± 0.036 ^c^
NaOH	12%	50.9 ± 0.53 ^a^	0.899 ± 0.060 ^d^	0.563 ± 0.025 ^c^	1.307 ± 0.144 ^c^	0.336 ± 0.036 ^d^	0.744 ± 0.167 ^c^
NaOH + Bleach	12%	51.2 ± 0.1 ^a,b^	0.069 ± 0.002 ^a^	0.089 ± 0.004 ^a^	0.121 ± 0.004 ^a^	−0.021 ± 0.002 ^b^	0.031 ± 0.002 ^b^
NaOH + Bleach	16%	51.7 ± 0.2 ^a,b^	0.270 ± 0.011 ^a^	0.369 ± 0.008 ^b^	0.421 ± 0.003 ^a^	−0.099 ± 0.005 ^b^	0.052 ± 0.006 ^b^
NaOH + Bleach	18%	50.3 ± 0.3 ^a^	0.558 ± 0.028 ^b^	0.728 ± 0.041 ^d^	0.748 ± 0.063 ^b^	−0.170 ± 0.026 ^a^	0.020 ± 0.044 ^b^
NaOH + Bleach	20%	50.0 ± 1.1 ^a^	0.872 ± 0.066 ^d^	1.116 ± 0.086 ^f^	0.732 ± 0.224 ^b^	−0.243 ± 0.055 ^a^	−0.383 ± 0.308 ^a^

Values are expressed as means of triplicates ± standard deviations. Data were subject to one-way analysis of variance (ANOVA) followed by Tukey’s post hoc multiple-comparison test. Means within same column followed by different superscript letters differ significantly (*p* < 0.05).

## Data Availability

The original contributions presented in this study are included in the article/Supplementary Materials. Further inquiries can be directed to the corresponding author.

## Supplementary Materials

The following supporting information can be downloaded at: https://www.mdpi.com/article/10.3390/polym18030356/s1, Table S1: Samples used in the experiments described in Section 2.3, Section 2.4, Section 2.5, Section 2.6, Section 2.7, Section 2.8, Section 2.9, Section 2.10, Section 2.11, Section 2.12, Section 2.13, Section 2.14, Section 2.15 and Section 2.16 of the Materials and Methods.; Figure S1: Representative report of aflatoxin strip test for spoiled chestnut flour. Tests were performed in triplicate according to the manufacturer’s instructions.; Figure S2: Representative report of aflatoxin strip test for NaHSO_3_ + Bleach starch. Tests were performed in triplicate according to the manufacturer’s instructions.; Figure S3: Representative report of aflatoxin strip test for NaOH + Bleach starch. Tests were performed in triplicate according to the manufacturer’s instructions.

## Abbreviations

The following abbreviations are used in this manuscript:

SEM	scanning electron microscopy
TGA	thermogravimetric analysis
DTG	derivative thermogravimetry
ESI-MS	electrospray ionization–mass spectrometry
HPSEC	high-performance size exclusion chromatography
MW	molecular weight
RI	refractive index
XRD	X-ray diffraction
%Cr	degree of crystallinity
ATR-FTIR	attenuated total reflectance–Fourier transform infrared spectroscopy
GT	gelatinization temperature
PV	peak viscosity
HPV	hot paste viscosity
CPV	cold paste viscosity
PV-HPV	breakdown
CPV-HPV	setback
%T	transmittance

## References

[1] Otero P., Echave J., Chamorro F., Soria-Lopez A., Cassani L., Simal-Gandara J., Prieto M.A., Fraga-Corral M. (2023). Challenges in the Application of Circular Economy Models to Agricultural By-Products: Pesticides in Spain as a Case Study. Foods.

[2] Sánchez-García E., Martínez-Falcó J., Marco-Lajara B., Manresa-Marhuenda E. (2024). Revolutionizing the circular economy through new technologies: A new era of sustainable progress. Environ. Technol. Innov..

[3] Campos D.A., Gomez-Garcia R., Vilas-Boas A.A., Madureira A.R., Pintado M.M. (2020). Management of Fruit Industrial By-Products-A Case Study on Circular Economy Approach. Molecules.

[4] Bigdeloo M., Teymourian T., Kowsari E., Ramakrishna S., Ehsani A. (2021). Sustainability and Circular Economy of Food Wastes: Waste Reduction Strategies, Higher Recycling Methods, and Improved Valorization. Mater. Circ. Econ..

[5] FAO Crops and Livestock Products. Food and Agriculture Organization of the United Nations (FAO). FAOSTAT 2023. https://www.fao.org/faostat/en/#data/QCL.

[6] APC VALORCAST—Valorização da Castanha e Optimização da Sua Comercialização. Associação Portuguesa da Castanha (APC). https://ec.europa.eu/eip/agriculture/en/find-connect/projects/valorcast-valoriza%C3%A7%C3%A3o-da-castanha-e-optimiza%C3%A7%C3%A3o-da.

[7] Zhu L., Jones C., Guo Q., Lewis L., Stark C.R., Alavi S. (2016). An evaluation of total starch and starch gelatinization methodologies in pelleted animal feed. J. Anim. Sci..

[8] Li H., Qi Y., Zhao Y., Chi J., Cheng S. (2019). Starch and its derivatives for paper coatings: A review. Prog. Org. Coat..

[9] Palanisamy A., Parimalavalli R. (2022). Resistant starch: A functional ingredient in dairy products. J. Food Process. Preserv..

[10] Homayouni A., Amini A., Keshtiban A.K., Mortazavian A.M., Esazadeh K., Pourmoradian S. (2013). Resistant starch in food industry: A changing outlook for consumer and producer. Starch-Stärke.

[11] Keenan M.J., Zhou J., McCutcheon K.L., Raggio A.M., Bateman H.G., Todd E., Jones C.K., Tulley R.T., Melton S., Martin R.J. (2006). Effects of resistant starch, a non-digestible fermentable fiber, on reducing body fat. Obesity.

[12] Montero B., Rico M., Barral L., Bouza R., López J., Schmidt A., Bittmann-Hennes B. (2021). Preparation and characterization of bionanocomposite films based on wheat starch and reinforced with cellulose nanocrystals. Cellulose.

[13] Dai L., Zhang J., Cheng F. (2019). Effects of starches from different botanical sources and modification methods on physicochemical properties of starch-based edible films. Int. J. Biol. Macromol..

[14] Correia P., Cruz-Lopes L., Beirão-da-Costa L. (2012). Morphology and structure of chestnut starch isolated by alkali and enzymatic methods. Food Hydrocoll..

[15] Correia P.R., Beirão-da-Costa M.L. (2012). Starch isolation from chestnut and acorn flours through alkaline and enzymatic methods. Food Bioprod. Process..

[16] Cruz B.R., Abraao A.S., Lemos A.M., Nunes F.M. (2013). Chemical composition and functional properties of native chestnut starch (*Castanea sativa* Mill). Carbohydr. Polym..

[17] Lemos A.M., Abraão A.S., Cruz B.R., Morgado M.L., Rebelo M., Nunes F.M. (2015). Effect of granular characteristics on the viscoelastic and mechanical properties of native chestnut starch (*Castanea sativa* Mill). Food Hydrocoll..

[18] Rafiq S.I., Jan K., Singh S., Saxena D.C. (2015). Physicochemical, pasting, rheological, thermal and morphological properties of horse chestnut starch. J. Food Sci. Technol..

[19] Yoo S.H., Lee C.S., Kim B.S., Shin M. (2012). The properties and molecular structures of gusiljatbam starch compared to those of acorn and chestnut starches. Starch-Stärke.

[20] Yang B., Jiang G., Prasad K.N., Gu C., Jiang Y. (2010). Crystalline, thermal and textural characteristics of starches isolated from chestnut (*Castanea mollissima* Bl.) seeds at different degrees of hardness. Food Chem..

[21] Bello-Pérez L.A., OrtÍz-Maldonado F., Villagómez-Mendez J., Toro-Vazquez J.F. (1998). Effect of Fatty Acids on Clarity of Starch Pastes. Starch-Stärke.

[22] Bello-Pérez L.A., Paredes-López O. (1996). Starch and Amylopectin—Effects of Solutes on Clarity of Pastes. Starch-Stärke.

[23] Zhang Y.-R., Wang X.-L., Zhao G.-M., Wang Y.-Z. (2012). Preparation and properties of oxidized starch with high degree of oxidation. Carbohydr. Polym..

[24] Broekman J.O.P., Genuino H.C., Heeres H.J., Brinksma J., Wielema T., Deuss P.J. (2023). Benign catalytic oxidation of potato starch using a homogeneous binuclear manganese catalyst and hydrogen peroxide. Catal. Sci. Technol..

[25] Chávez-Murillo C.E., Wang Y., Bello-Pérez L.A. (2008). Morphological, Physicochemical and Structural Characteristics of Oxidized Barley and Corn Starches. Starch-Stärke.

[26] Kuakpetoon D., Wang Y.-J. (2001). Characterization of Different Starches Oxidized by Hypochlorite. Starch-Stärke.

[27] Soliman A.A.A., EI-Shinnawy N.A., Mobarak F. (1997). Thermal behaviour of starch and oxidized starch. Thermochim. Acta.

[28] Lin Z., Xia Y., Yang G., Chen J., Ji D. (2019). Improved film formability of oxidized starch-based blends through controlled modification with cellulose nanocrystals. Ind. Crops Prod..

[29] Zhang Y., Li G., Wu Y., Yang Z., Ouyang J. (2019). Influence of amylose on the pasting and gel texture properties of chestnut starch during thermal processing. Food Chem..

[30] Yu Y., Wang Y.N., Ding W., Zhou J., Shi B. (2017). Preparation of highly-oxidized starch using hydrogen peroxide and its application as a novel ligand for zirconium tanning of leather. Carbohydr. Polym..

[31] Dang X., Chen H., Wang Y., Shan Z. (2018). Freeze-drying of oxidized corn starch: Electrochemical synthesis and characterization. Cellulose.

[32] Simões J., Nunes F.M., Domingues M.R., Coimbra M.A. (2011). Demonstration of the presence of acetylation and arabinose branching as structural features of locust bean gum galactomannans. Carbohydr. Polym..

[33] Huang J., Schols H., Vansoest J., Jin Z., Sulmann E., Voragen A. (2007). Physicochemical properties and amylopectin chain profiles of cowpea, chickpea and yellow pea starches. Food Chem..

[34] Srichuwong S., Sunarti T.C., Mishima T., Isono N., Hisamatsu M. (2005). Starches from different botanical sources II: Contribution of starch structure to swelling and pasting properties. Carbohydr. Polym..

[35] DuBois M., Gilles K.A., Hamilton J.K., Rebers P.A., Smith F. (1956). Colorimetric Method for Determination of Sugars and Related Substances. Anal. Chem..

[36] Chrastil J. (1987). Improved colorimetric determination of amylose in starches or flours. Carbohydr. Res..

[37] Achille T.F., Georges A.N., Alphonse K. (2007). Contribution to light transmittance modeling in starch media. Afr. J. Biotechnol..

[38] Whistler R.L., Linke E.G., Kazeniac S. (1956). Action of alkaline hypochlorite on corn starch amylose and methyl 4-O-methyl-d-glucopyranoside. J. Am. Chem. Soc..

[39] European Commission (2023). COMMISSION REGULATION (EU) 2023/915 of 25 April 2023 on maximum levels for certain contaminants in food and repealing Regulation (EC) No 1881/2006. Off. J. Eur. Union.

[40] Zhou F., Liu Q., Zhang H., Chen Q., Kong B. (2016). Potato starch oxidation induced by sodium hypochlorite and its effect on functional properties and digestibility. Int. J. Biol. Macromol..

[41] Boruch M. (1985). Transformations of Potato Starch During Oxidation with Hypochlorite. Starch-Stärke.

[42] Fukuzaki S. (2006). Mechanisms of Actions of Sodium Hypochlorite in Cleaning and Disinfection Processes. Biocontrol Sci..

[43] Patel K.F., Mehta H.U., Srivastava H.C. (1974). Kinetics and mechanism of oxidation of starch with sodium hypochlorite. J. Appl. Polym. Sci..

[44] Rutenberg M.W., Solarek V., Chapter X., Whistler R.L., Bemiller J.N., Paschall E.F. (1984). Starch derivatives: Production and uses. Starch: Chemistry and Technology.

[45] Liu X., Yu L., Xie F., Li M., Chen L., Li X. (2010). Kinetics and mechanism of thermal decomposition of cornstarches with different amylose/amylopectin ratios. Starch-Stärke.

[46] Elomaa M. (2004). Determination of the degree of substitution of acetylated starch by hydrolysis, 1H NMR and TGA/IR. Carbohydr. Polym..

[47] Bogracheva T.Y., Wang Y.L., Wang T.L., Hedley C.L. (2002). Structural studies of starches with different water contents. Biopolymers.

[48] van Soest J.J.G., Tournois H., de Wit D., Vliegenthart J.F.G. (1995). Short-range structure in (partially) crystalline potato starch determined with attenuated total reflectance Fourier-transform IR spectroscopy. Carbohydr. Res..

[49] Capron I., Robert P., Colonna P., Brogly M., Planchot V. (2007). Starch in rubbery and glassy states by FTIR spectroscopy. Carbohydr. Polym..

[50] Miao M., Zhang T., Mu W., Jiang B. (2010). Effect of controlled gelatinization in excess water on digestibility of waxy maize starch. Food Chem..

