# Supplementary material for: The Oxidative Extraction of Starch from Chestnut (Castanea sativa Mill.) Byproducts: A Valorization Strategy for a Sustainable Food Industry
Source: Polymers (Basel). 2026 Jan 28;18(3):356. doi: 10.3390/polym18030356 (PMC12899577; doi:10.3390/polym18030356)
Supplement: Supplementary file 1 [file polymers-18-00356-s001.zip › polymers-4127004-supplementary.pdf]

Supplementary Material

The Oxidative Extraction of Starch from Chestnut (*Castanea sativa* Mill.) Byproducts: A Valorization Strategy for a Sustainable Food Industry

Table S1. The samples used in the experiments described in Sections 2.3- 2.16 of the Materials and Methods.

2.3. Color evaluation	2.4. SEM	2.5. TGA	2.6. Aflatoxin analysis	2.7. Total starch	2.8. Carboxyl content 2.9. Carbonyl content	2.10. ESI-MS	2.11. HPSEC	2.12. XRD	2.13. ATR-FTIR	2.14. Swelling power, starch solubility, and leached amylose	2.15. Pasting properties	2.16. Transmittance
												Potato starch
Commercial corn starch										Commercial corn starch		Commercial corn starch
Conforming chestnut flour		Conforming chestnut flour						Conforming chestnut flour				
Spoiled chestnut flour		Spoiled chestnut flour	Spoiled chestnut flour					Spoiled chestnut flour				
Conforming chestnut starch_$NaHSO_3$		Conforming chestnut starch_$NaHSO_3$		Conforming chestnut starch_$NaHSO_3$		Conforming chestnut starch_$NaHSO_3$	Conforming chestnut starch_$NaHSO_3$	Conforming chestnut starch_$NaHSO_3$	Conforming chestnut starch_$NaHSO_3$	Conforming chestnut starch_$NaHSO_3$	Conforming chestnut starch_$NaHSO_3$	Conforming chestnut starch_$NaHSO_3$
Conforming chestnut starch_$NaHSO_3$												

(from flour)												
Spoiled chestnut starch_$NaHSO_3$	Spoiled chestnut starch_$NaHSO_3$	Spoiled _chestnut_starch_$NaHSO_3$					Spoiled chestnut starch_$NaHSO_3$	Spoiled chestnut starch_$NaHSO_3$				
Spoiled chestnut starch_$NaHSO_3$+Bleach	Spoiled chestnut starch_$NaHSO_3$ +Bleach	Spoiled chestnut starch $NaHSO_3$+Bleach	Spoiled chestnut starch $NaHSO_3$+ Bleach	Spoiled chestnut starch $NaHSO_3$+ Bleach	Spoiled chestnut starch $NaHSO_3$+ Bleach	Spoiled chestnut starch $NaHSO_3$+Bleach	Spoiled chestnut starch $NaHSO_3$ +Bleach	Spoiled chestnut starch $NaHSO_3$+Bleach	Spoiled chestnut starch $NaHSO_3$+Bleach	Spoiled chestnut starch $NaHSO_3$+Bleach	Spoiled chestnut starch $NaHSO_3$+Bleach	Spoiled chestnut starch $NaHSO_3$+Bleach
Conforming chestnut starch_NaOH		Conforming chestnut starch_NaOH		Conforming chestnut starch_NaOH		Conforming chestnut starch_NaOH	Conforming chestnut starch_NaOH	Conforming chestnut starch_NaOH	Conforming chestnut starch_NaOH	Conforming chestnut starch_NaOH	Conforming chestnut starch_NaOH	Conforming chestnut starch_NaOH
Conforming chestnut starch_NaOH (from flour)												
Spoiled chestnut starch_NaOH	Spoiled chestnut starch_NaOH	Spoiled _chestnut_starch_NaOH					Spoiled chestnut starch_NaOH	Spoiled chestnut starch_NaOH				
Spoiled chestnut	Spoiled chestnut	Spoiled chestnut	Spoiled chestnut	Spoiled chestnut	Spoiled chestnut	Spoiled chestnut	Spoiled chestnut	Spoiled chestnut	Spoiled chestnut	Spoiled chestnut	Spoiled chestnut	Spoiled chestnut

starch_NaOH+Bleach	starch NaOH+ Bleach	starch NaOH+Bleach	starch NaOH+Bleach	starch NaOH+Bleach	starch NaOH+Bleach	starch NaOH+Bleach	starch NaOH+ Bleach	starch NaOH+Bleach	starch NaOH+Bleach	starch NaOH+Bleach	starch NaOH+Bleach	starch NaOH+Bleach

Sample Report

Date generated	Wed, 14 Jan 2026 16:37
User	ambifoodridasmart@gmail.com

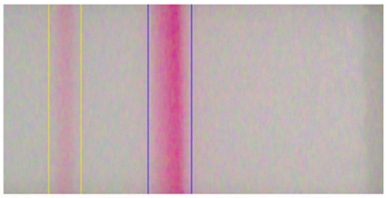

Result	< 2 ppb
Sample ID	Chestnut flour_2
Supplier	
Date	Wed, 14 Jan 2026 16:34
Dilution Factor	1.0
Product	RIDA QUICK Aflatoxin RQS
Application	Methanol Corn 2 - 75 ppb
Art. No.	R5208x
Lot	24245
Notes	

Figure S1. Representative report of aflatoxin strip test for spoiled chestnut flour. Tests were performed in triplicate according to manufacturer's instructions.

Sample Report

Date generated	Wed, 14 Jan 2026 14:36
User	ambifoodridasmart@gmail.[illegible]

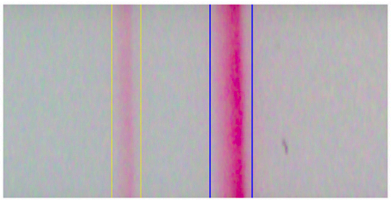

Result	< 2 ppb
Sample ID	NaHSO3_1
Supplier	
Date	Wed, 14 Jan 2026 14:31
Dilution Factor	1.0
Product	RIDA QUICK Aflatoxin RQS
Application	Methanol Corn 2 - 75 ppb
Art. No.	R5208x
Lot	24245
Notes	

Figure S2. Representative report of aflatoxin strip test for $NaHSO_3$+Bleach starch. Tests were performed in triplicate according to manufacturer's instructions.

Sample Report

Date generated	Wed, 14 Jan 2026 15:59
User	ambifoodridasmart@gmail.com

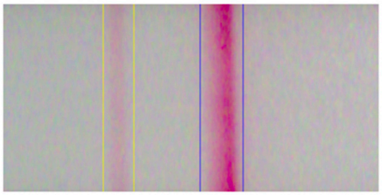

Result	< 2 ppb
Sample ID	NaOH_1
Supplier	
Date	Wed, 14 Jan 2026 15:57
Dilution Factor	1.0
Product	RIDA QUICK Aflatoxin RQS
Application	Methanol Corn 2 - 75 ppb
Art. No.	R5208x
Lot	24245
Notes	

Figure S3. Representative report of aflatoxin strip test for NaOH+Bleach starch. Tests were performed in triplicate according to manufacturer's instructions.
